# Efficacy and safety of traditional Chinese medicine (TCM) combined with immune checkpoint inhibitors (ICIs) for the treatment of cancer: a systematic review and meta-analysis

**DOI:** 10.3389/fphar.2025.1661503

**Published:** 2025-10-31

**Authors:** Yani Ke, Yuyan Pan, Xueru Huang, Xing Bai, Xiaojuan Liu, Mingsi Zhang, Yunhai Wei, Tao Jiang, Guangji Zhang

**Affiliations:** ^1^ School of Basic Medical Sciences, Zhejiang Chinese Medical University, Hangzhou, Zhejiang, China; ^2^ Key Laboratory of Blood-stasis-toxin Syndrome of Zhejiang Province, Hangzhou, China; ^3^ Traditional Chinese Medicine “Preventing Disease” Wisdom Health Project Research Center of Zhejiang, Hangzhou, China; ^4^ The First Affiliated Hospital of Zhejiang Chinese Medical University (Zhejiang Province Hospital of Chinese Medicine), Hangzhou, Zhejiang, China; ^5^ Huzhou Central Hospital, Huzhou, Zhejiang, China

**Keywords:** TCM, traditional Chinese medicine, ICIS, immune checkpoint inhibitors, cancer, meta-analysis

## Abstract

**Background:**

Cancer remains a major global health burden. Combining traditional Chinese medicine (TCM) with immune checkpoint inhibitors (ICIs) may potentially mitigate treatment-related side effects and improve the quality of life for cancer patients. To critically evaluate the clinical efficacy of this combination therapy, a meta-analysis was performed.

**Methods:**

A systematic search was conducted across six databases. Data were analyzed using RevMan 5.3 and Stata 12. Heterogeneity was explored through subgroup analysis and meta-regression. The robustness of results was assessed via sensitivity analysis and publication bias.

**Results:**

41 studies were included. The TCM + ICIs group demonstrated significantly superior outcomes compared to the ICIs group across multiple endpoints: Overall Response Rate (ORR) (RR: 1.34 [1.20, 1.49]), Disease Control Rate (DCR) (RR: 1.15 [1.10, 1.21]), CD4^+^/CD8^+^ T-cell ratio (WMD: 0.25 [0.15, 0.35]), Progression-Free Survival (PFS) (WMD: 0.96 [0.29, 1.63]), Overall Survival (OS) (WMD: 1.46 [0.62, 2.30]), Karnofsky Performance Status (KPS) (WMD: 6.35 [4.99, 7.70]), and TCM Therapeutic Evaluation (RR: 1.42 [1.30, 1.55]). Conversely, the TCM + ICIs group showed lower levels of tumor markers, including Alpha-Fetoprotein (AFP) (SMD: 0.75 [-1.49, −0.01]), Carcinoembryonic Antigen (CEA) (SMD: 0.72 [-1.08, −0.37]), Carbohydrate Antigen 125 (CA125) (SMD: 0.77 [-1.46, −0.08]), and a reduced incidence of adverse events (RR: 0.82 [0.69, 0.97]). There is high heterogeneity among CD4+T/CD8+T studies due to the type of tumor and whether it is combined with chemotherapy. The high heterogeneity among studies on KPS may be related to the type of ICIs. Sensitivity analysis and assessment of publication bias confirmed the robustness of the pooled results.

**Conclusion:**

The combination of TCM with ICIs appears to enhance antitumor immunity, reduce adverse reactions, lower serum tumor marker levels, improve disease control, and ameliorate patient performance status. This combination strategy represents a promising therapeutic approach for various cancers and warrants further investigation.

## Introduction

Cancer poses a significant global health challenge, exerting a profound impact on human life. According to Global Cancer Observatory (GCO) data, there were approximately 20 million new cancer cases and nearly 10 million cancer-related deaths worldwide in 2022. Projections indicate that by 2050, new cancer cases could exceed 35 million annually (Bray et al., 2024). Lung, liver, stomach, breast, and colon cancers are among the leading causes of cancer mortality (Cao et al., 2021). Current treatment modalities include local therapies such as surgical resection, radiotherapy, interventional therapy, and ablation, as well as systemic therapies like chemotherapy. However, these approaches often have limitations and can be associated with substantial side effects (Zeng, 2018).

Recent years have witnessed remarkable advances in cancer immunotherapy. Immune checkpoint inhibitors (ICIs), particularly monoclonal antibodies targeting programmed cell death protein 1 (PD-1), its ligand (PD-L1), and cytotoxic T-lymphocyte-associated antigen 4 (CTLA-4), have become first-line treatments for various malignancies, significantly improving survival outcomes for many patients (Bagchi et al., 2021; Cunningham et al., 2024). ICIs function by blocking inhibitory immune signals, thereby potentiating T cell-mediated anti-tumor responses (Brahmer et al., 2021). Despite their efficacy, the widespread clinical application of ICIs has revealed a spectrum of immune-related adverse events (irAEs), such as myocarditis, pneumonitis, and hepatitis. The increasing incidence of these toxicities, along with the emergence of drug resistance, presents significant clinical challenges (Hu et al., 2022; Wang Z. et al., 2023).

Accumulating evidence suggests that various forms of Traditional Chinese Medicine (TCM) can inhibit the proliferation and metastasis of diverse tumor cells. TCM has shown notable benefits in treating cancers such as breast, lung, liver, and gastric cancer, potentially extending survival, improving quality of life, and enhancing the efficacy while reducing the toxicity of combined radiotherapy and chemotherapy (Wang K. et al., 2021; Xiang et al., 2019). Preclinical and clinical studies have begun to verify the synergistic effects of TCM and ICIs in cancers including lung cancer, breast cancer, and melanoma. Potential underlying mechanisms include modulation of the tumor microenvironment and regulation of gut microbiota (Yu YX. et al., 2023). Consequently, the combination of TCM and ICIs is gaining acceptance, although the precise mechanisms of action remain incompletely elucidated. This meta-analysis aims to clarify the efficacy and potential mechanisms of the TCM + ICIs combination in cancer treatment, with the goal of informing clinical practice and providing new perspectives for therapeutic development.

## Methods

### Literature search

The protocol for this study has been registered on the PROSPERO website (https://www.crd.york.ac.uk/prospero/) with number CRD42024582055 in Additional File 1. Two independent investigators systematically searched six databases (PubMed, Embase, Cochrane Library, CNKI, Wanfang, and CBM) for literature published up to 7 October 2024. The search strategy combined Medical Subject Headings (MeSH) terms with free words, incorporating key concepts such as ‘Traditional Chinese Medicine’ (TCM), ‘immune checkpoint inhibitors’, ‘PD-1′, ‘PD-L1′, and ‘ICIs’. The detailed search strategy is provided in Additional File 2. Relevant references from the retrieved articles were also screened for inclusion. The entire process adhered strictly to the PRISMA guidelines (Moher et al., 2009) (see Additional File 3). Botanical drugs included in this study were verified using the Medicinal Plant Names Services (MPNS) portal (http://mpns.kew.org/mpns-portal/), while other traditional medicines were authenticated via the Zhong Hui Zhong Yao Wang database (https://www.zhzyw.com/).

### Study selection

Two reviewers independently screened the titles and abstracts of retrieved records, followed by full-text assessment for eligibility. A third reviewer resolved any discrepancies and made final judgments based on the pre-defined protocol. The specific inclusion criteria were as follows:

All included literature must meet the following: 1) Study participants were clinically diagnosed cancer patients aged 18 years or older, with both an experimental and a control group; 2) The intervention consisted of ICIs combined with TCM, which could include TCM monomers, single herbs, herb pairs, prescriptions, or moxibustion/fumigation therapies; 3) The control group received ICIs for cancer; 4) Reported outcomes included at least one of the following: Overall Response Rate (ORR), Disease Control Rate (DCR), CD4+/CD8+ T-cell ratio, Progression-Free Survival (PFS), Overall Survival (OS), Karnofsky Performance Status (KPS), levels of Alpha-Fetoprotein (AFP), Carcinoembryonic Antigen (CEA), Carbohydrate Antigen 125 (CA125), Carbohydrate Antigen 19-9 (CA199), incidence of adverse effects, or TCM therapeutic evaluation; 5) Study design was a randomized controlled trial (RCT) comparing ICI combination therapy with TCM versus ICI monotherapy. Concurrent conventional or basic supportive care was permitted in both groups. Common ICIs considered included Pembrolizumab, Nivolumab, Sintilimab, Tislelizumab, Camrelizumab, and so on.

### Data extraction and quality assessment

Data extraction and quality assessment were performed independently by two reviewers. A third reviewer was consulted to reconcile disagreements and finalize the data synthesis. Extracted data encompassed: publication year, corresponding author’s country/region, cancer diagnostic criteria, baseline characteristics of participants (sample size, age, gender), details of interventions (types, dosage, administration of ICIs; types, formulation, administration of TCM; other concomitant treatments; treatment duration), and all pre-specified outcomes (ORR, DCR, CD4+/CD8+ ratio, PFS, OS, KPS, AFP, CEA, CA125, CA19-9, adverse effects, TCM evaluation). Corresponding authors were contacted for missing or unclear data. Furthermore, ORR was defined as the proportion of patients achieving a complete response (CR) or partial response (PR). DCR was defined as the proportion of patients achieving CR, PR, or stable disease (SD) (Upadhyay et al., 2025; Bae et al., 2022). Both ORR and DCR are key metrics for tumor response evaluation.

The methodological quality of the included RCTs was assessed using the Cochrane Risk of Bias tool (RoB 2.0) (Higgins et al., 2011). This tool evaluates six domains: i) randomization process, ii) allocation concealment, iii) blinding of participants and personnel, iv) blinding of outcome assessment, v) incomplete outcome data, and vi) selective reporting. Each domain was judged as ‘low risk of bias’, ‘some concerns’, or ‘high risk of bias’. To enhance the reporting transparency and reproducibility of the included TCM interventions, the ConPhyMP tool was referenced, following the approach of Heinrich et al.

### Statistical analysis

Data analysis was conducted using RevMan (version 5.3) and Stata (version 12). For dichotomous outcomes, data were pooled and expressed as Risk Ratios (RR) with 95% confidence intervals (CI). Continuous outcomes were categorized based on their distribution. Data following a normal distribution were presented as mean ± standard deviation (SD). Non-normally distributed data were converted to mean ± SD using established methods (Shi et al., 2023; Luo et al., 2018; Wan et al., 2014) via https://www.math.hkbu.edu.hk/∼tongt/pages/median2mean.html. The Weighted Mean Difference (WMD) and 95% CI were applied when outcomes were measured on the same unit across studies; otherwise, the Standardized Mean Difference (SMD) and 95% CI were used. Heterogeneity among studies was quantitatively assessed using the I^2^ statistic and Cochran’s Q test, with results visualized using forest plots. An I^2^ value less than 50% indicated low heterogeneity, warranting the use of a fixed-effects model. An I^2^ value greater than 50% suggested substantial heterogeneity, leading to the adoption of a random-effects model (Schmidt et al., 2009; Higgins et al., 2003). If sufficient studies were available (n > 10), subgroup analysis or meta-regression was planned to explore potential sources of heterogeneity. Sensitivity analysis was performed by sequentially excluding each study to evaluate the robustness of the pooled results. Publication bias was assessed using Egger’s test (Egger et al., 1997) when more than five studies were included in a meta-analysis. A p-value <0.05 indicated potential publication bias, in which case the trim-and-filling method was employed to assess their stability.

## Results

### Study selection

**FIGURE 1:**
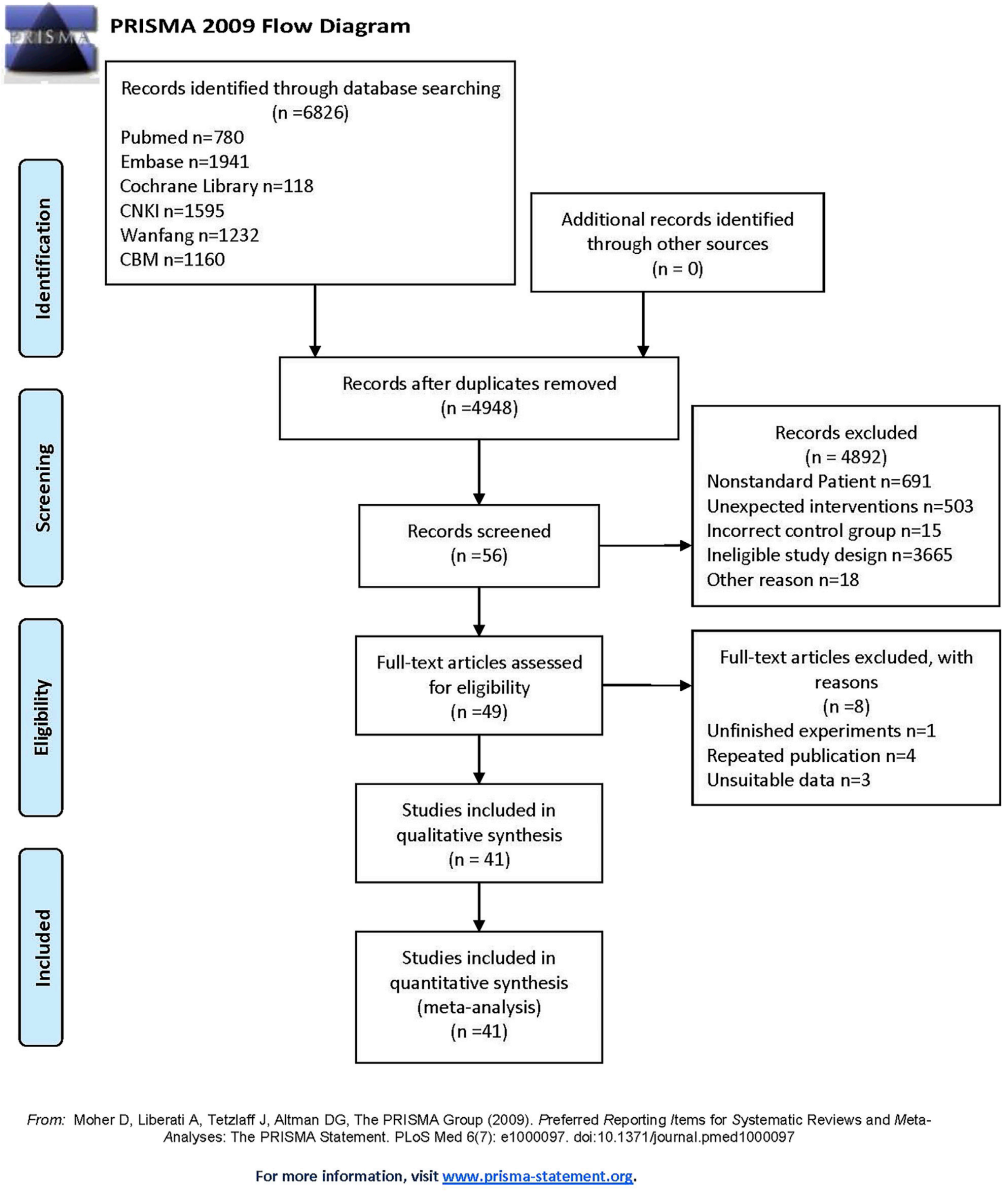
Flowchart of study inclusions and exclusions.

A total of 6,826 articles were retrieved from the six databases. After removal of duplicates, 4,948 articles remained for screening by two independent reviewers based on the inclusion and exclusion criteria. Ultimately, 41 studies (Chai, 2023; Chen, 2022; Ding et al., 2024; Du and Liu, 2022; Fang, 2023; Jiang and Fang, 2024; Lin et al., 2022; Lin, 2023; Liu et al., 2022; Luo, 2023; Qu, 2023; Wang L. et al., 2023; Wang Q., 2023; Wang et al., 2024; Wang XH., 2023; Wang, 2021; Xiao et al., 2022; Yang, 2023; Yao, 2023; Zhang J., 2023; Zhang, 2022; Zhao et al., 2023; Zhao, 2024; Zhou, 2023; Zhu, 2024; Cao et al., 2024; Cao, 2023; Huang et al., 2024; Ye and Fang, 2022; Yu D. et al., 2023; Zhang LL., 2023; Zhong, 2023; Dou, 2022; Liu and Wang, 2024; Liu and Xia, 2024; Ye, 2022; Huang et al., 2023; Wang, 2024; Xu, 2023; Zhang et al., 2023; Zhou and Xie, 2024) were included in the meta-analysis. All included studies were conducted in China, with only three published in English databases. This analysis encompassed 2,612 cancer patients, including cases of lung cancer (n = 1,631), liver cancer (n = 490), gastric cancer (n = 154), esophageal cancer (n = 227), colorectal cancer (n = 50), and ovarian cancer (n = 60). Various ICIs were used, such as Sintilimab, Nivolumab, Camrelizumab, and Tislelizumab. TCM interventions included prescriptions, moxibustion, injections, and other formulations. The publication years of the included studies ranged from 2020 to 2024, indicating a focus on recent research. The characteristics of the included studies are summarized in Table 1, and the study selection flow diagram is presented in Figure 1. The TCM included in each study are detailed in Additional File 4, with all drugs having been verified.

**TABLE 1:** Baseline characteristics of studies included.

No.	Author	Year	Country/Region	Trial period	No. of patients		Age		Therapeutic measure		Types of cancer	Indicators
					TCM + ICIs group	ICIs group	TCM + ICIs group	ICIs group	TCM + ICIs group	ICIs group		
1	Chai (2023)	2023	China	2020.1–2022.12	30	30	\	\	Carilizumab + Yiqi tongluo jiedu decoction	Carilizumab	Lung cancer	ORR, DCR, CEA, CA125, Adverse Effects, TCM Therapeutic evaluation
2	Chen (2022)	2022	China	2020.10–2022.3	30	30	60.13 ± 12.40	64.67 ± 9.28	Carilizumab + Astragalus Injection	Carilizumab	Lung cancer	ORR, DCR, Adverse effects
3	Ding et al. (2024)	2023	China	2020.12–2022.12	36	36	54.39 ± 10.93	56.03 ± 12.22	Multiple PD-1+Zhigancao decoction	Multiple PD-1	Lung cancer	ORR, CEA, KPS
4	Du and Liu (2022)	2022	China	2018.5–2020.5	41	41	54.74 ± 8.03	55.53 ± 7.98	Nivolumab + Jianpi bufei formula	Nivolumab	Lung cancer	ORR, DCR, CD4+T/CD8+T, Adverse effects
5	Fang (2023)	2023	China	2021.10–2022.11	21	22	68.91 ± 7.26	67.9 ± 6.79	Multiple PD-1+Modified bazhen decoction	Multiple PD-1	Lung cancer	CD4+T/CD8+T, TCM Therapeutic evaluation
6	Jiang and Fang (2024)	2024	China	2020.3–2022.5	43	42	55.25 ± 5.18	56.14 ± 5.22	Nivolumab + Buzhong yiqi Decoction	Nivolumab	Lung cancer	ORR, DCR, CD4+T/CD8+T, PFS, OS
7	Lin et al. (2022)	2022	China	2020.10–2021.5	20	20	57.9 ± 9.6	59.5 ± 10.6	Nivolumab + Fuzheng jiandu formula	Nivolumab	Lung cancer	ORR, DCR
8	Lin (2023)	2023	China	2021.12–2022.12	30	30	60.5 (51.75, 64)	60.5 (56.5, 63)	Carilizumab + Shenqi fuzheng injection	Carilizumab	Lung cancer	ORR, DCR, CD4+T/CD8+T
9	Liu et al. (2022)	2022	China	2019.2–2021.2	28	25	41.63 ± 12.57	43.47 ± 11.63	Nivolumab + Xiaoyan decoction	Nivolumab	Lung cancer	ORR, CD4+T/CD8+T, KPS
10	Luo (2023)	2023	China	2019.6–2021.6	44	44	69.34 ± 7.81	69.21 ± 7.86	Nivolumab + Jianpi bufei formula	Nivolumab	Lung cancer	ORR, DCR, CD4+T/CD8+T
11	Qu (2023)	2023	China	2022.1–2023.2	32	30	65.86 ± 4.08	64.78 ± 4.35	Xindilimumab + Bufei decoction	Xindilimumab	Lung cancer	ORR, DCR, KPS, TCM Therapeutic evaluation
12	Wang et al. (2023b)	2023	China	2020.2–2022.2	44	44	64.33 ± 3.55	64.37 ± 3.27	Carilizumab + Jianpi huatan xiaoying decoction	Carilizumab	Lung cancer	Adverse effects
13	Wang (2023a)	2023	China	2021.12–2022.12	32	31	65.54 ± 10.39	66.91 ± 10.36	Tislelizumab + Modified liujunzi decoction	Tislelizumab	Lung cancer	ORR, DCR, CD4+T/CD8+T, KPS, TCM Therapeutic evaluation
14	Wang et al. (2024)	2024	China	2021.10–2022.5	29	30	68.58 ± 8.4	69.06 ± 5.44	Tislelizumab + Qingfei tiaoqi decoction	Tislelizumab	Lung cancer	Adverse effects
15	Wang (2023b)	2023	China	2020.11–2022.11	30	31	66.13 ± 6.26	65.48 ± 5.59	Tislelizumab + Dushen decoction	Tislelizumab	Lung cancer	ORR, DCR, CD4+T/CD8+T, PFS, TCM Therapeutic evaluation
16	Wang, (2021)	2021	China	2019.5–2020.11	30	30	64.07 ± 4.83	63.74 ± 4.528	Nivolumab + Shenqi yifei decoction	Nivolumab	Lung cancer	CD4+T/CD8+T, CEA, CA125
17	Xiao et al. (2022)	2022	China	2021.3–2022.3	25	25	74.15 ± 2.01	74.21 ± 2.02	Pembrolizumab + TCM decoction	Pembrolizumab	Lung cancer	ORR, DCR, Adverse effects
18	Yang (2023)	2023	China	2020.7–2022.7	30	28	63.12 ± 10.94	64.31 ± 6.67	Multiple ICIs + Yanghe decoction	Multiple ICIs	Lung cancer	CEA, CA125
19	Yao (2023)	2023	China	2021.12–2022.12	25	25	64.80 ± 7.15	65.04 ± 9.88	Xindilimumab + Qigui buxue syrup	Xindilimumab	Lung cancer	CD4+T/CD8+T, TCM Therapeutic evaluation
20	Zhang (2023a)	2023	China	2021.1–2022.9	33	32	68.61 ± 8.10	68.72 ± 7.73	PD-1+Shenqi fuzheng injection	PD-1	Lung cancer	ORR, DCR, Adverse effects
21	Zhang (2022)	2022	China	2020.12–2021.12	30	30	63.06 ± 3.503	63.50 ± 3.048	Xindilimumab + Jianpi chuji formula	Xindilimumab	Lung cancer	ORR, DCR, KPS, TCM Therapeutic evaluation
22	Zhao et al. (2023)	2023	China	2019.1–2021.1	50	50	62.45 ± 5.24	61.93 ± 5.36	Pembrolizumab + Peitu zishen formula	Pembrolizumab	Lung cancer	ORR, DCR
23	Zhao (2024)	2024	China	2021.12–2022.12	30	30	60.30 ± 6.924	58.53 ± 8.378	Xindilimumab + Yiqi shengmai formula moxibustion	Xindilimumab	Lung cancer	ORR, DCR, CD4+T/CD8+T, KPS, TCM Therapeutic evaluation
24	Zhou (2023)	2023	China	2021.9–2022.12	45	45	59.95 ± 7.72	57.33 ± 7.65	Xindilimumab + Shenqi busui decoction	Xindilimumab	Lung cancer	KPS, TCM Therapeutic evaluation
25	Zhu (2024)	2024	China	2022.12–2024.2	31	31	\	\	Multiple ICIs + Fuzheng guben formula	Multiple ICIs	Lung cancer	ORR, DCR, CEA, TCM Therapeutic evaluation
26	Cao et al. (2024)	2024	China	2021.10–2023.07	40	40	42.9 ± 10.5	42.8 ± 10.7	Xindilimumab + Wenyang fuzheng decoction	Xindilimumab	Liver cancer	CD4+T/CD8+T, KPS, AFP, TCM Therapeutic evaluation
27	Cao (2023)	2023	China	2021.12–2022.12	28	27	60.13 ± 12.40	64.67 ± 9.28	Carilizumab + Yangzheng xiaoji capsules	Carilizumab	Liver cancer	ORR, DCR, CD4+T/CD8+T, TCM Therapeutic evaluation
28	Huang et al. (2024)	2023	China	2020.1–2022.12	30	30	65.9 ± 8.8	66.6 ± 12.4	Xindilimumab + Sini decoction	Xindilimumab	Liver cancer	AFP, KPS, Adverse effects
29	Ye and Fang (2022)	2022	China	2019.9–2021.11	20	20	61.50 ± 4.32	62.10 ± 7.06	Carilizumab + Jianpi huoxue formula	Carilizumab	Liver cancer	ORR, DCR, CD4+T/CD8+T,AFP, CEA, KPS, TCM Therapeutic evaluation
30	Yu et al. (2023b)	2023	China	2018.1–2022.6	56	56	46.27 ± 6.87	45.76 ± 6.28	Multiple ICIs + Ruyi jinhuang powder	Multiple ICIs	Liver cancer	ORR, DCR, AFP, CA199, KPS, Adverse effects
31	Zhang (2023b)	2023	China	2021.4–2022.12	22	21	\	\	Multiple ICIs + Liver cancer formula I	Multiple ICIs	Liver cancer	ORR, DCR, Adverse effects
32	Zhong (2023)	2023	China	2021.1–2022.12	50	50	53.4 ± 6.2	54.7 ± 7.8	Carilizumab + Fuhe beihua formula	Carilizumab	Liver cancer	ORR, DCR, PFS, AFP, KPS
33	Dou (2022)	2022	China	2020.1–2022.1	30	30	64.87 ± 6.11	65.7 ± 6.95	Carilizumab + Jianpi huatan quyu formula	Carilizumab	Esophageal cancer	ORR, DCR, TCM Therapeutic evaluation
34	Liu and Wang (2024)	2024	China	2020.3–2022.3	19	24	\	\	Carilizumab + Xiaoaiping injection	Carilizumab	Esophageal cancer	CEA, CA125, CA199
35	Liu and Xia (2024)	2024	China	2022.6–2023.6	30	30	60.33 ± 8.75	57.93 ± 5.40	Carilizumab + Fuzheng sanjie formula	Carilizumab	Esophageal cancer	ORR, DCR
36	Ye (2022)	2022	China	2021.1–2021.12	32	32	67.12 ± 10.87	69.56 ± 10.70	Carilizumab + Modified wumei decoction	Carilizumab	Esophageal cancer	ORR, DCR
37	Huang et al. (2023)	2023	China	2020.1–2021.12	25	26	69.44 ± 11.78	69.92 ± 9.81	Multiple PD-1+WD-3	Multiple PD-1	Gastric cancer	ORR, DCR, KPS
38	Wang (2024)	2024	China	2022.4–2023.12	22	23	65.91 ± 7.43	62.43 ± 13.08	Xindilimumab + Jianpi huatan formula	Xindilimumab	Gastric cancer	ORR, DCR, KPS, TCM Therapeutic evaluation
39	Xu (2023)	2023	China	2021.1–2022.10	29	29	\	\	Xindilimumab + Wenyang tongluo formula	Xindilimumab	Gastric cancer	ORR, DCR, CD4+T/CD8+T, PFS, OS, TCM Therapeutic evaluation
40	Zhang et al. (2023)	2023	China	2020.1–2021.12	25	25	56. 9 ± 7. 6	58. 1 ± 6. 5	Penpulimab + Bushen jiedu sanjie formula	Penpulimab	Colorectal cancer	ORR, DCR, CD4+T/CD8+T
41	Zhou and Xie (2024)	2024	China	2021.6–2023.6	30	30	\	\	Carilizumab + Guizhi fuling pill	Carilizumab	Ovarian cancer	ORR, DCR, CA125

TCM, traditional chinese medicine; ICIs, Immune Checkpoint Inhibitors; PD-1, Programmed Cell Death Protein 1; ORR, overall response rate; DCR, disease control rate; PFS, Progression-Free Survival; OS, overall survival; KPS, karnofsky; AFP, Alpha-Fetoprotein; CEA, carcinoembryonic antigen; CA125, Carbohydrate antigen 125; CA199, Carbohydrate antigen 199.

### Quality assessment and data extraction

**FIGURE 2:**
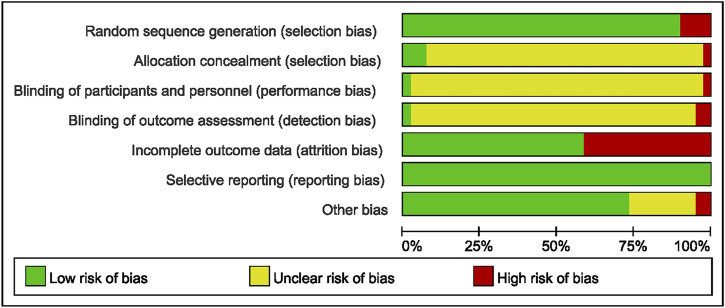
Risk of bias analysis across studies: Risk of bias item presented as a percentage across all included trials (n = 41).

The methodological quality of the 41 included studies, assessed using the Cochrane RoB 2.0 tool, is summarized in Figure 2 and detailed in Additional File 5. Most studies reported random allocation of participants to the case or control group; however, only two studies specified the involvement of a third researcher in the randomization process. Blinding procedures were seldom mentioned, leading to potential performance and detection bias. Several studies reported participant dropouts, with reasons for attrition not always being consistent between groups. All studies adhered to their pre-specified outcome measures without selective reporting. Overall, the quality of the included studies was variable, with some demonstrating robust methodology and others having unclear reporting of design elements. The assessment of TCM preparation reporting quality using the ConPhyMP tool is provided in Additional Files 6 and 7.

### TCM + ICIs vs. ICIs in cancer—Overall Response Rate (ORR)

**FIGURE 3:**
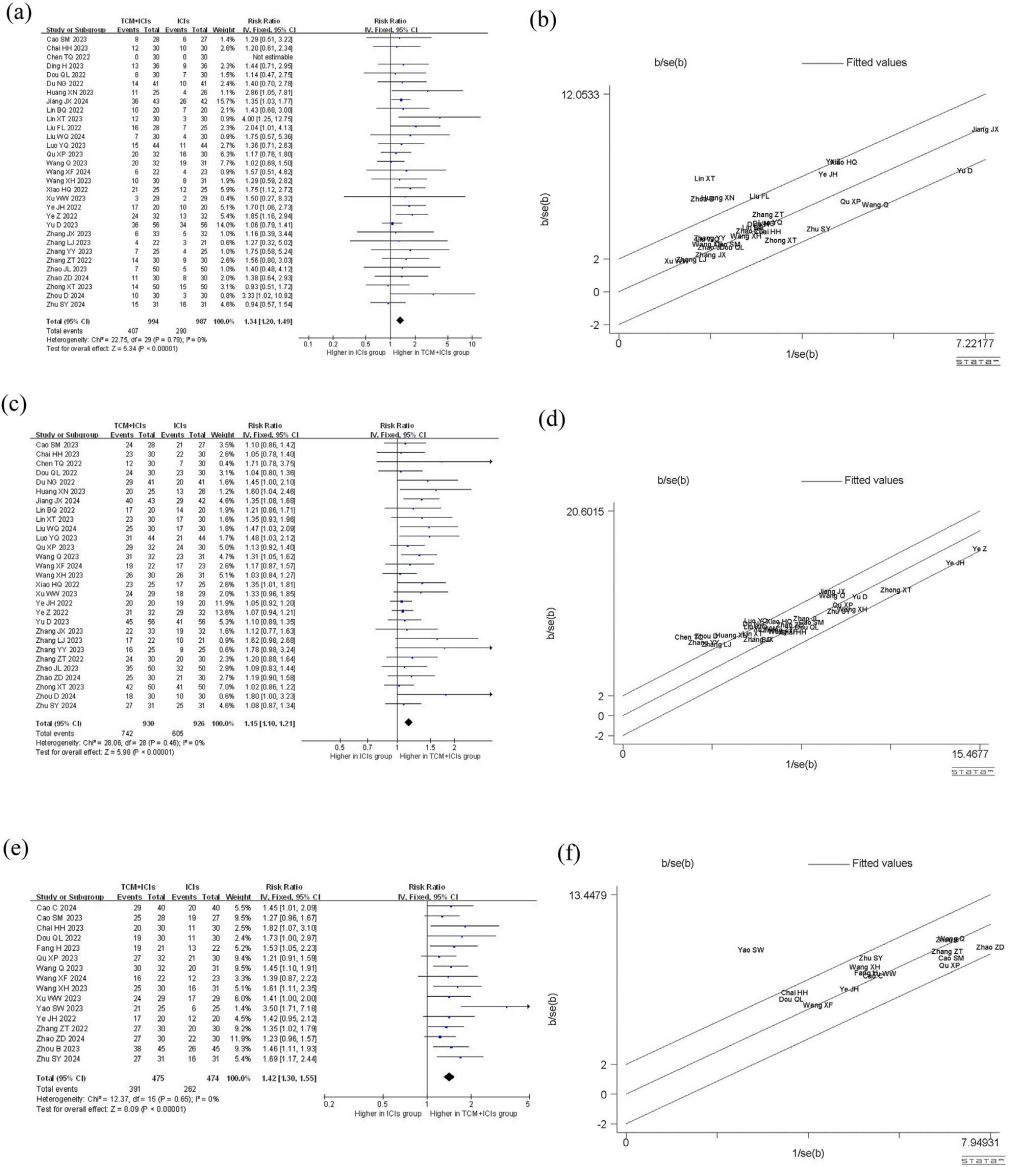
Meta-analysis and Galbr analysis of TCM + ICIs vs. ICIs in cancer—ORR, DCR, TCM Therapeutic Evaluation: **(a)** Meta-analysis of TCM + ICIs vs. ICIs in cancer—ORR; **(b)** Galbr analysis of TCM + ICIs vs. ICIs in cancer—ORR; **(c)** Meta-analysis of TCM + ICIs vs. ICIs in cancer—DCR; **(d)** Galbr analysis of TCM + ICIs vs. ICIs in cancer—DCR; **(e)** Meta-analysis of TCM + ICIs vs. ICIs in cancer—TCM Therapeutic Evaluation; **(f)** Galbr analysis of TCM + ICIs vs. ICIs in cancer—TCM Therapeutic Evaluation.

The comparison of ORR between two groups of patients is shown in Figure 3. The heterogeneity among 31 studies was low (p = 0.79, I^2^ = 0%), therefore a fixed-effect model was chosen. The results showed that the patients in TCM + ICIs group had higher ORR values (RR = 1.34 [1.20–1.49]) compared to those in ICIs group, with RR = 1 as the reference. In addition, exploration on different cancers were also conducted (see Table 2), and the results showed that the TCM + ICIs group had higher ORR values in lung cancer, gastric cancer, esophageal cancer, and other tumors (RR = 1.37 [1.19,1.58], RR = 2.07 [1.05,4.08], RR = 1.63 [1.10,2.41], RR = 2.43 [1.09,5.40], respectively).

**TABLE 2:** Subgroup analysis of TCM + ICIs vs. ICIs in cancer. (A)ORR; (B)DCR; (C)TCM Therapeutic evaluation.

Study or subgroup	Data sets	TCM + ICIs group		ICIs group		Weight	Risk ratio	P	I^2^
		Events	Total	Events	Total		M-H, fixed, 95%CI		
ORR^A^									
Lung cancer	18	252	595	181	588	62.6	1.37 [1.19, 1.58]	0.8	0%
Liver cancer	5	79	176	68	174	23.40%	1.16 [0.92, 1.45]	0.49	0%
Gastric cancer	3	20	76	10	78	3.40%	2.07 [1.05, 4.08]	0.68	0%
Esophageal cancer	3	39	92	24	92	8.20%	1.63 [1.10, 2.41]	0.63	0%
Others	2	17	55	7	55	2.40%	2.43 [1.09, 5.40]	0.43	0%
Total	31	407	994	290	987	100.00%	1.39 [1.24, 1.56]	0.77	0%
DCR^B^									
Lung cancer	16	417	531	337	527	55.80%	1.23 [1.14, 1.32]	0.77	0%
Liver cancer	5	148	176	132	174	21.90%	1.11 [1.00, 1.23]	0.46	0%
Gastric cancer	3	63	76	48	78	7.80%	1.35 [1.10, 1.65]	0.47	0%
Esophageal cancer	3	80	92	69	92	11.40%	1.16 [1.01, 1.33]	0.14	49%
Others	2	34	55	19	55	3.10%	1.79 [1.18, 2.72]	0.98	0%
Total	29	742	930	605	926	100.00%	1.22 [1.16, 1.29]	0.22	16%
TCM Therapeutic Evaluation^C^									
Lung cancer	10	261	306	171	305	65.30%	1.52 [1.36, 1.69]	0.19	28%
Liver cancer	3	71	88	51	87	19.60%	1.37 [1.12, 1.68]	0.81	0%
Gastric cancer	2	40	51	29	52	10.90%	1.40 [1.06, 1.86]	0.97	0%
Others	1	19	30	11	30	4.20%	1.73 [1.00, 2.97]	—	—
Total	16	391	475	262	474	100.00%	1.49 [1.36, 1.63]	0.56	0%

ORR, overall response rate; DCR, disease control rate; TCM, traditional chinese medicine; ICIs, Immune Checkpoint Inhibitors; CI, Confidence Interval.

### TCM + ICIs vs. ICIs in cancer—Disease Control Rate (DCR)

There are a total of 29 studies involving DCR, and the results of meta-analysis and heterogeneity analysis are shown in Figure 3. The fixed-effect model was applied (p = 0.46, I^2^ = 0%). The results showed that the DCR values of TCM + ICIs group were significantly higher than those of ICIs group, with RR 1.15 (1.10, 1.21). The subgroup analysis results of different cancers also demonstrated that regardless of the type of cancer, patients in TCM + ICIs group have higher DCR in Table 2.

### TCM + ICIs vs. ICIs in cancer—Cd4+T/CD8+T

**FIGURE 4:**
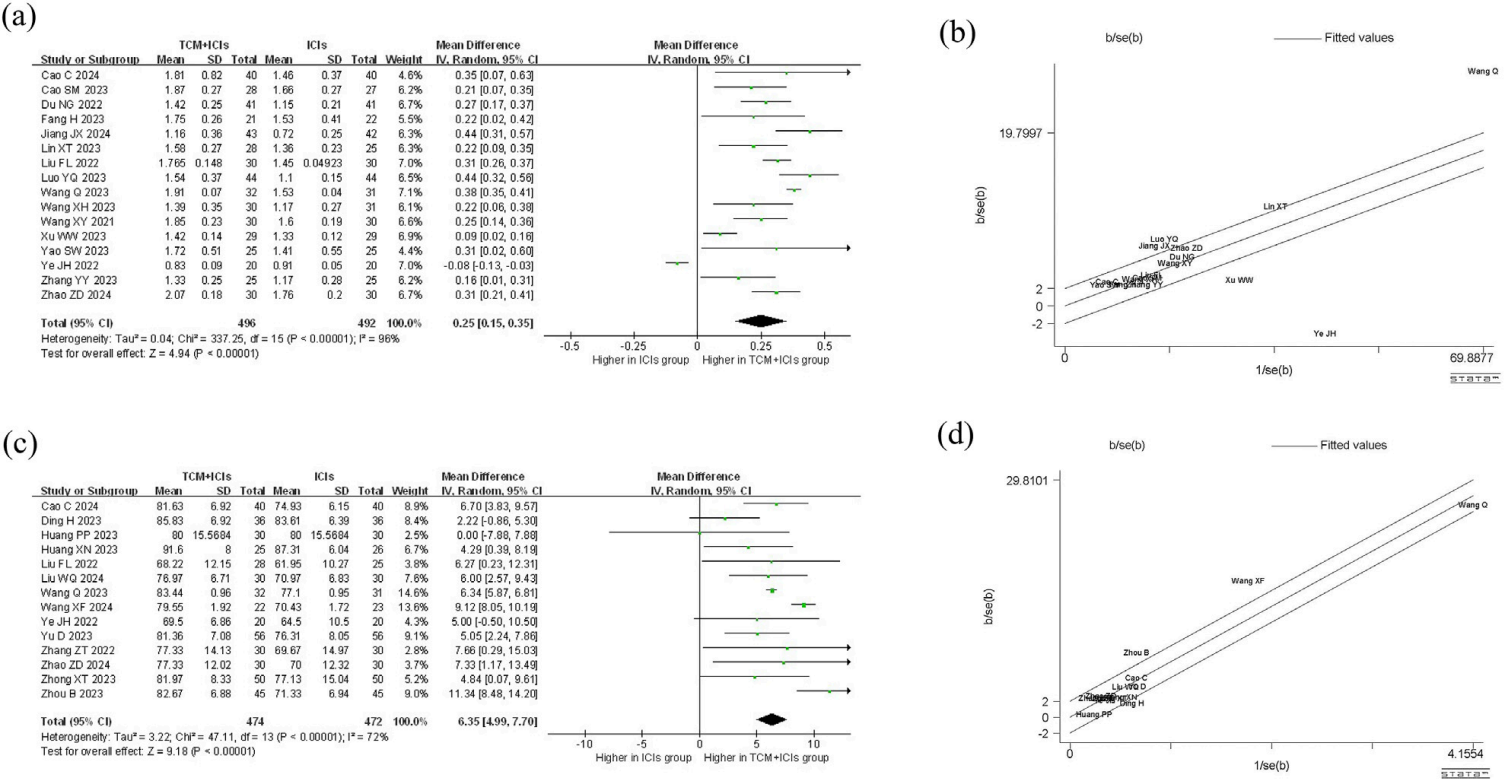
Meta-analysis and Galbr analysis of TCM + ICIs vs. ICIs in cancer—CD4+T/CD8+T or KPS: **(a)** Meta-analysis of TCM + ICIs vs. ICIs in cancer—CD4+T/CD8+T; **(b)** Galbr analysis of TCM + ICIs vs. ICIs in cancer—CD4+T/CD8+T; **(c)** Meta-analysis of TCM + ICIs vs. ICIs in cancer—KPS; **(d)** Galbr analysis of TCM + ICIs vs. ICIs in cancer—KPS.

The meta-analysis and heterogeneity analysis of 16 studies are shown in Figure 4. There is obvious heterogeneity (p < 0.05, I^2^ = 96%), so the random-effect model is the best choice. The CD4+T/CD8+T ratio in TCM + ICIs group was significantly higher than that in ICIs group, with WMD 0.25 (0.15, 0.35). Due to the presence of high heterogeneity, subgroup analysis and meta-regression were performed to further explore its sources. The results in Table 3 showed that heterogeneity among subgroups decreased when grouped based on different cancers or ICIs, but it cannot be determined whether it is the source of heterogeneity. Therefore, the results of meta-regression are particularly important. The results in Table 4 suggest that whether it is lung cancer and whether both groups receive chemotherapy regimens may be sources of high heterogeneity (p < 0.05). Unfortunately, further multi-meta-regression was not performed.

**TABLE 3:** Subgroup analysis of TCM + ICIs vs. ICIs in cancer—CD4+T/CD8+T by different factors. (A) Different kinds of cancers; (B) Different kinds of ICIs; (C) Different combination therapies.

Study or subgroup	Data sets	Model	TCM + ICIs group	ICIs group	Weight	Mean difference	P	I2
			Total	Total		95%CI		
Different kinds of cancers^A^								
Lung cancer	11	—	354	351	69.10%	0.32 [0.27, 0.37]	0.007	59%
Liver cancer	3	—	88	87	17.80%	0.14 [-0.13, 0.40]	<0.00001	91%
Other cancer	2	—	54	54	13.10%	0.10 [0.04, 0.16]	0.4	0%
Total	16	Random	496	492	100.00%	0.25 [0.15, 0.35]	<0.00001	96%
Different kinds of ICIs^B^								
Nivolumab	5	—	186	182	32.40%	0.32 [0.23, 0.41]	0.02	66%
Xindilimumab	4	—	124	124	22.60%	0.24 [0.08, 0.40]	0.001	81%
Carilizumab	3	—	78	77	20.20%	0.15 [-0.15, 0.44]	<0.00001	99%
Trelizumab	2	—	62	62	13.10%	0.32 [0.17, 0.47]	0.05	74%
Others	2	—	46	47	11.70%	0.18 [0.06, 0.30]	0.64	0%
Total	16	Random	496	492	100.00%	0.25 [0.15, 0.35]	<0.00001	96%
Different combination therapies^C^								
Chemotherapy	11	—	353	350	66.60%	0.28 [0.21, 0.36]	<0.00001	87%
Targeted therapy	3	—	77	76	18.9%	0.06 [-0.09, 0.22]	<0.00001	93%
No other therapies	3	—	95	95	14.5%	0.27 [0.17, 0.36]	0.77	0%
Total	16	Random	525	521	100.0%	0.24 [0.15, 0.34]	<0.00001	96%

TCM, traditional chinese medicine; ICIs, Immune Checkpoint Inhibitors; CI, Confidence Interval.

**TABLE 4:** Meta-regression of TCM + ICIs vs. ICIs in cancer—CD4+T/CD8+T.

Covariates	Data sets	Coefficient	Standard error	t	P	95%CI
Univariate meta-regression analysis						
Lung cancer or not	16	1.244	0.071	3.81	0.002	[1.100, 1.407]
Types of ICIs	16	1.091	0.081	1.17	0.261	[0.930, 1.279]
Chemotherapy combined or not	16	1.178	0.076	2.54	0.023	[1.026, 1.353]

ICIs, Immune Checkpoint Inhibitors; CI, Confidence Interval.

### TCM + ICIs vs. ICIs in cancer—Progression-Free Survival (PFS)/Overall Survival (OS)

Table 5 contains a comparison of PFS and OS between two groups of patients. The meta-analysis of PFS used a random-effect model (p < 0.05, I^2^ = 91%), and the results showed that the PFS of TCM + ICIs group was significantly higher than that of ICIs group (WMD = 0.96 [0.29, 1.63]). Due to the small number of studies included (n = 4), heterogeneity exploration was not conducted. Meanwhile, only two studies involved OS, and the heterogeneity among them was not high (p = 0.48, I^2^ = 0%). After applying the fixed-effect model, the results showed that TCM + ICIs group had a longer OS period, with WMD 1.46 (0.62, 2.30).

**TABLE 5:** Meta-analysis of TCM + ICIs vs. ICIs in cancer.

Content	Data sets	Model	WMD/SMD/RR	P	I^2^
PFS	4	Random	WMD: 0.96 [0.29, 1.63]	<0.00001	91%
OS	2	Fixed	WMD: 1.46 [0.62, 2.30]	0.48	0%
AFP	4	Random	SMD: 0.75 [-1.49, −0.01]	<0.00001	89%
CEA	7	Random	SMD: 0.72 [-1.08, −0.37]	0.007	66%
CA125	5	Random	SMD: 0.77 [-1.46, −0.08]	<0.00001	87%
CA199	2	Random	SMD: 0.95 [-2.68, 0.78]	<0.00001	95%
Adverse Event	12	Random	RR: 0.82 [0.69, 0.97]	0.0005	67%

WMD, weighted mean difference; RR, relative risk; SMD, standardized mean difference; ORR, overall response rate; DCR, disease control rate; PFS, Progression-Free Survival; OS, overall survival; KPS, karnofsky; AFP, Alpha-Fetoprotein; CEA, carcinoembryonic antigen; CA125, Carbohydrate antigen 125; CA199, Carbohydrate antigen 199.

### TCM + ICIs vs. ICIs in cancer—Karnofsky Performance Status (KPS)

14 studies are related to KPS, and there is significant heterogeneity among them (p < 0.05, I^2^ = 72%). As shown in Figure 4, the results of the random-effect model demonstrate that the KPS score of TCM + ICIs group is significantly higher than that of the ICIs group, with WMD 6.35 (4.99, 7.70). The Galbr plot also showed high heterogeneity among studies. Thus, subgroup analysis and meta-regression were also performed. Through subgroup analysis of different cancers and ICIs, heterogeneity of each group has decreased, shown in Table 6, suggesting that they may be the source of heterogeneity (Table 7). Univariate meta-regression suggests that different types of ICIs are indeed sources of high heterogeneity (p < 0.05), which deserves further research.

**TABLE 6:** Subgroup analysis of TCM + ICIs vs. ICIs in cancer—KPS by different factors. (A) Different kinds of cancers; (B) Different kinds of ICIs.

Study or subgroup	Data sets	Model	TCM + ICIs group	ICIs group	Weight	Mean difference	P	I^2^
			Total	Total		95%CI		
Different kinds of cancers^A^								
Lung cancer	6	—	201	197	42.10%	6.77 [4.17, 9.38]	0.002	73%
Liver cancer	5	—	196	196	30.00%	5.37 [3.66, 7.08]	0.6	0%
Other cancer	3	—	77	79	27.90%	6.90 [3.75, 10.04]	0.02	74%
Total	14	Random	474	472	100.00%	6.35 [4.99, 7.70]	<0.00001	72%
Different kinds of ICIs^B^								
Xindilimumab	6	—	197	198	40.30%	8.30 [6.25, 10.35]	0.06	52%
Carilizumab	3	—	100	100	17.20%	5.48 [3.00, 7.96]	0.91	0%
Mixed	3	—	117	118	24.10%	3.88 [2.05, 5.71]	0.4	0%
Others	2	—	60	56	18.30%	6.34 [5.87, 6.81]	0.98	0%
Total	14	Random	474	472	100.00%	6.35 [4.99, 7.70]	<0.00001	72%

KPS, karnofsky performance status; TCM, traditional chinese medicine; ICIs, Immune Checkpoint Inhibitors; CI, Confidence Interval.

**TABLE 7:** Meta-regression of TCM + ICIs vs. ICIs in cancer—KPS.

Covariates	Data sets	Coefficient	Standard error	t	P	95%CI
Univariate meta-regression analysis						
Lung cancer or not	14	2.41	3.883	0.55	0.595	[0.072, 80.647]
Types of ICIs	14	27.497	30.403	3	0.011	[2.472, 305.878]

ICIs, Immune Checkpoint Inhibitors; CI, Confidence Interval.

### TCM + ICIs vs. ICIs in cancer—Alpha-Fetoprotein (AFP)

Only four studies involved AFP (Table 5), and there was significant heterogeneity (p < 0.05, I^2^ = 89%). After the application of the random-effect model, the AFP level in TCM + ICIs group was significantly lower than that in ICIs group, with SMD -0.75 (- 1.49, − 0.01). The source of heterogeneity has not been explored.

### TCM + ICIs vs. ICIs in cancer—Carcinoembryonic Antigen (CEA)/carbohydrate antigen 125 (CA125)/carbohydrate antigen 199 (CA199)

Nine studies are included in this section (Table 5), including seven on CEA, five on CA125, and two on CA199. Due to the presence of high heterogeneity, they all adopted a random-effect model (p < 0.05, I^2^ = 66%; p < 0.05, I^2^ = 87%; p < 0.05, I^2^ = 95%, respectively). All results demonstrated that the levels of CEA and CA125 in TCM + ICIs group were significantly lower than those in ICIs group (SMD = −0.72 [-1.08, −0.37]; SMD = −0.77 [-1.46, −0.08], respectively), and there was no significant difference in CA199 levels between the two groups (SMD = −0.95 [-2.68, 0.78]). However, the number of studies included in this section is limited, which also affects the reliability of the conclusion.

### TCM + ICIs vs. ICIs in cancer—Adverse event

The results of the adverse events of the two groups are presented in Table 5. There was high heterogeneity among the 12 included studies (p = 0.0005, I^2^ = 67%), therefore a random-effect model was selected. The incidence of adverse events in TCM + ICIs group was significantly lower than that in ICIs group (RR = 0.82 [0.69, 0.97]). Further analysis was conducted based on different adverse events, including gastrointestinal reactions, myelosuppression, hypertension, thyroid dysfunction, liver dysfunction and kidney dysfunction. The final results showed that the various adverse reactions in TCM + ICIs group were significantly lower than those in ICIs group shown in Table 8.

**TABLE 8:** Subgroup analysis of TCM + ICIs vs. ICIs in cancer by different adverse events.

Study or subgroup	Data sets	TCM + ICIs group		ICIs group		Weight	Risk ratio	P	I^2^
		Events	Total	Events	Total		M-H, fixed, 95%CI		
Gastrointestinal reactions	13	100	436	180	435	39.80%	0.55 [0.46, 0.66]	0.03	48%
Myelosuppression	7	40	196	82	198	18.00%	0.49 [0.37, 0.66]	0.88	0%
Hypertension	6	33	217	59	216	13.10%	0.56 [0.38, 0.81]	0.64	0%
Thyroid dysfunction	11	29	323	48	321	10.80%	0.60 [0.39, 0.93]	0.81	0%
Liver dysfunction	13	39	458	60	455	13.70%	0.66 [0.46, 0.94]	0.75	0%
Kidney dysfunction	6	8	204	20	202	4.60%	0.41 [0.19, 0.88]	0.67	0%

TCM, traditional chinese medicine; ICIs, Immune Checkpoint Inhibitors; CI, confidence interval.

### TCM + ICIs vs. ICIs in cancer—TCM therapeutic evaluation

Figure 3 shows the TCM Therapeutic Evaluation of two groups, which includes 16 related studies. Due to insignificant heterogeneity (p = 0.65, I^2^ = 0%), a fixed-effect model was chosen. The overall TCM therapeutic evaluation of TCM + ICIs group was higher than that of ICIs group, with RR 1.42 (1.30, 1.55). Regardless of the type of cancer, the TCM therapeutic evaluation of TCM + ICIs group was significantly increased shown in Table 2.

### Sensitivity analysis

The results of all sensitivity analyses can be found in Additional File 8. The stability of each meta-analysis can be determined by whether the changes of the results are significant or whether there is a reversal after removing each study one by one. After comprehensive evaluation, it was found that all 11 meta-analyses in this study have good stability (with no significant changes in the results).

### Publication bias

Only meta-analyses with more than 5 included studies were evaluated for publication bias. The publication bias results of these seven meta-analyses were based on Eggar’s Test (Additional File 9). When it comes to ORR, DCR, CD4+T/CD8+T, Adverse Event, and TCM therapeutic evaluation, significant publication bias cannot be ignored (p < 0.05), while the other two meta-analyses are not. Therefore, the trim-and-filling method was executed to further evaluate whether the presence of publication bias affects the reliability and robustness of the results. This adjustment did not substantially alter or reverse the original pooled results, supporting the robustness and representativeness of the findings despite the presence of publication bias.

## Discussion

The immune system plays a critical role in anti-tumor defense by identifying and eliminating mutated cells through immune surveillance, thereby preventing tumor development (Gao et al., 2022). Cancer immunotherapy represents a milestone in oncology, shifting the therapeutic focus from solely targeting malignant cells to modulating the tumor microenvironment (Rui et al., 2023). ICIs, a class of immunotherapy, function by reactivating T cells in the tumor microenvironment and enhancing natural killer (NK) cell activity. This process prevents tumor immune escape and facilitates the conversion of immunologically “cold” tumors to “hot” tumors (Zhang et al., 2022). ICIs are monoclonal antibodies targeting inhibitory checkpoint molecules expressed on antigen-presenting cells (APCs) and CD4^+^ T cells (Marei et al., 2023). Currently, FDA-approved ICIs include CTLA-4 inhibitors (e.g., ipilimumab), PD-1 inhibitors (e.g., nivolumab, pembrolizumab, cemiplimab), and PD-L1 inhibitors (e.g., atezolizumab, durvalumab, avelumab) (Vafaei et al., 2022). By blocking ligand-receptor interactions on T cells, ICIs reverse immunosuppression and inhibit tumor growth (Andrews et al., 2019). Evidence indicates that ICIs enhance anti-tumor immunity by modulating the PD-1/PD-L1 and CTLA-4/CD80/86 pathways, strengthening tumor antigen recognition and ultimately inducing tumor cell death (Seto et al., 2019). However, ICIs can disrupt peripheral self-tolerance, triggering autoimmune-like inflammatory responses known as immune-related adverse events (irAEs) (Zhou et al., 2024). These irAEs may involve multiple organ systems, such as cardiac, endocrine, gastrointestinal, dermatologic, and renal, and can lead to significant inflammation and visceral toxicity (Ramos-Casals et al., 2022). Identifying strategies to mitigate the incidence and severity of irAEs remains a key research priority.

In China, Traditional Chinese Medicine (TCM) represents a major therapeutic modality for cancer treatment. TCM derives from three primary sources: botanical, animal, and microbial materials. Its therapeutic effects are not attributable to isolated drugs but arise from the synergistic interactions among drug combinations and complex interactions, producing multi-target regulatory activities that collectively exert anti-tumor effects. Numerous studies have confirmed that various traditional Chinese medicines and their main metabolites can inhibit tumor progression, alleviate radiotherapy- and chemotherapy-induced side effects, and improve survival in cancer patients (Li et al., 2012). Proposed anti-tumor mechanisms of TCM include the suppression of cancer cell proliferation, migration, and invasion, promotion of tumor vascular normalization, and interference with metastatic processes (Cheng et al., 2021; Chen et al., 2022). A growing body of clinical and preclinical evidence supports the combined use of TCM with immunotherapy, demonstrating that such combinations can enhance efficacy, reverse drug resistance, and reduce adverse reactions (Zhang et al., 2020). It was confirmed through *in vivo* and *in vitro* experiments that bufonidin can reverse the activation of phosphatidylinositol 3-kinase (PI3K)/(Serine/Threonine Kinase B) AKT/(mammalian target of rapamycin) mTOR signaling pathway induced by bifunctional apoptosis regulator (BFAR), and enhance the efficacy of combination with ICIs (Chen G. et al., 2023). Cordycepin, combined with anti–CTLA-4 therapy, alleviated CD8^+^ T cell exhaustion in the tumor microenvironment by upregulating chemokine expression (Chen L. et al., 2023). Similarly, the combination of evodiamine and anti–PD-1 therapy more effectively suppressed Lewis lung cancer growth by increasing CD8^+^ T cell infiltration in blood, tumor, and spleen, reducing Treg proportions, and promoting the secretion of cytokines such as Tumor Necrosis Factor-alpha (TNF-α), Recombinant Granzyme B (GZMB), and Interferon-gamma (IFN-γ) (Jiang et al., 2020). These findings collectively indicate that TCM and ICIs can act synergistically, suggesting a promising approach for enhancing cancer treatment outcomes.

This meta-analysis evaluated 41 randomized controlled trials investigating the efficacy of TCM combined with ICIs in cancer treatment. All included studies were conducted in China and published within the past 5 years. Although most reported random allocation, blinding procedures were rarely described. The results showed that the ORR, DCR, CD4+T/CD8+T, PFS, OS, KPS, adverse events, and TCM therapeutic evaluation of the TCM + ICIs group were significantly higher than those of the ICIs group. On the contrary, the AFP, CEA, and CA125 levels in TCM + ICIs group were significantly lower than those in ICIs group, while CA199 showed no significant difference. These findings suggest that TCM combination therapy may enhance immune function, improve performance status, reduce tumor marker levels, and ameliorate treatment-related toxicity. However, the efficacy of TCM + ICIs appears to vary by cancer type. The present analysis primarily included studies on respiratory and digestive tract tumors. For respiratory cancers, TCM + ICIs consistently outperformed ICIs monotherapy. Among gastrointestinal cancers, effects were more heterogeneous; notably, in liver cancer, no significant differences were observed in ORR, DCR, or CD4+/CD8+ ratio between groups, whereas benefits were evident in other digestive malignancies. These discrepancies may reflect the limited number of available studies or differential tumor biology and TCM sensitivity, highlighting the need for further clinical validation. Furthermore, the overall incidence of adverse reactions and the incidence of adverse reactions in each system in TCM + ICIs group were lower than those in ICIs group. From the perspective of modern pharmacological research, some active metabolites in TCM can exert a synergistic effect with immunosuppressants to reduce the occurrence of adverse reactions. TCM can affect enzyme activity, regulating the metabolic rate of immunosuppressants in the body, avoiding drug accumulation, and reducing adverse reactions such as liver and kidney damage (Wang S. et al., 2021). Meanwhile, it can alleviate excessive inflammation caused by immunosuppressants and reduce inflammation related tissue damage by relying on its own anti-inflammatory and oxygen free radical scavenging effects (Zhu et al., 2022). In addition, the protective effect of TCM on organs can weaken the direct stimulation and damage of immunosuppressants, thereby reducing adverse reactions (Ren et al., 2021).

The heterogeneity among studies on ORR, DCR, OS, and TCM therapeutic evaluation is not high, so fixed-effect models have been well applied. However, significant heterogeneity was observed in other outcome analyses, necessitating investigation into its sources. Given the varying number of studies across outcomes, subgroup analysis and meta-regression were only feasible for selected endpoints. When CD4^+^T/CD8^+^T is grouped based on tumor type and ICIs type, the heterogeneity among different subgroups decreases. The conclusion drawn from meta-regression is that whether it is lung cancer and whether chemotherapy treatment is applied may both be sources of heterogeneity, suggesting that TCM treatment may be more effective for lung cancer and the possible clinical efficacy of TCM with immunotherapy and chemotherapy. During the subgroup analysis of KPS, the heterogeneity among subgroups of different tumors or ICIs was significantly reduced. Further meta-regression analysis shows that the source of heterogeneity is the type of ICIs. These findings suggest that TCM’s therapeutic effects may vary by tumor type and ICI agent. Tumor-specific pathophysiology and ICI mechanism may influence TCM compatibility and efficacy. Currently, TCM + ICIs show consistent benefit in lung cancer across ICI types. As ICI diversity and indication breadth continue to expand, future research should clarify which ICIs are most suitable for specific patient subgroups and baseline characteristics, enabling treatment personalization and optimized resource use. If more systematic and comprehensive research can be conducted in the later stage, it is an essential part of improving clinical efficacy and avoiding resource waste. The reliability and robustness of the results are crucial for drawing correct conclusions. Sensitivity analysis confirmed the stability of all meta-analyses. However, Egger’s test indicated potential publication bias for ORR, DCR, CD4^+^/CD8^+^T, adverse events, and TCM therapeutic evaluation. Subsequent trim-and-filling analysis confirmed that the pooled results remained robust despite such bias, underscoring the clinical relevance of this study.

As the first meta-analysis to assess the clinical efficacy of TCM combined with ICIs for cancer, this review has several limitations. First, the number of included studies remains limited, with strong representation of lung cancer but relatively few trials on other common malignancies such as liver or gastric cancer. Second, all studies were conducted in China; although they cover multiple regions, potential ethnic variations in treatment response cannot be ruled out. As numerous preclinical studies support TCM’s anti-tumor properties, international clinical trials are warranted to assess the generalizability of TCM + ICI therapy. Third, in the quality assessment, most studies were rated as having “unclear” risk of bias across several domains, which may compromise the overall credibility of the evidence and limit its direct clinical applicability. Finally, although this meta-analysis evaluated multiple efficacy endpoints, incomplete reporting in many publications constrained deeper subgroup analysis. It is suggested that future clinical studies should adopt more comprehensive and standardized reporting to facilitate more precise evidence synthesis.

## Conclusion

Compared with ICIs monotherapy, the combination of TCM and ICIs significantly improves ORR, DCR, CD4+/CD8+ T-cell ratio, PFS, OS, KPS, and TCM therapeutic evaluation, while reducing levels of AFP, CEA, and CA125 and the incidence of adverse events. Subgroup analysis and meta-regression revealed that heterogeneity in CD4+/CD8+ T-cell ratio was influenced by tumor type and concomitant chemotherapy, while heterogeneity in KPS was associated with ICI type. Combining sensitivity analysis, publication bias analysis, and the trim-and-filling method, the results of our studies are robust and reliable. However, our research is still limited in terms of quantity and region, and we hope more clinicians and researchers will engage in this field to contribute higher-quality data and more comprehensive insights.

## Data Availability

The original contributions presented in the study are included in the article/Supplementary Material, further inquiries can be directed to the corresponding authors.

## Glossary

**GCO**: Global Clinical Operations
**ICIs**: Immune Checkpoint Inhibitors
**PD-1**: Programmed Cell Death Protein 1
PD-L1: Programmed Death Ligand 1
**CTLA-4**: Cytotoxic T Lymphocyte Antigen-4
**TCM**: Traditional Chinese Medicine
**ORR**: Overall Response Rate
**DCR**: Disease Control Rate
**PFS**: Progression-Free Survival
**OS**: Overall Survival
KPS: Karnofsky Performance Status
**AFP**: Alpha-Fetoprotein
**CEA**: Carcinoembryonic Antigen
CA125: Carbohydrate antigen 125
**CA199**: Carbohydrate antigen 199
**CR**: Complete remission
**PR**: Partial remission
SD: Stable disease
RR: Relative Risk
**WMD**: Weighted Mean Difference
**CI**: Confidence Intervals
**SMD**: Standardized Mean Difference
**mAb**: Monoclonal Antibody
**APC**: Antigen Presenting Cells
FDA: Food and Drug Administration
**irAEs**: immune related Adverse Events
PI3K: Phosphatidylinositol 3-kinase
**AKT**: Serine/Threonine Kinase B
**mTOR**: mammalian target of rapamycin
**BFAR**: Bifunctional Apoptosis Regulator
**TME**: Tumor Microenvironment
**TNF-α**: Tumor Necrosis Factor-alpha
**GZMB**: Recombinant Granzyme B
**IFN-γ**: Interferon-gamma

## References

[B1] Andrews L. P. Yano H. Vignali D. A. A. (2019). Inhibitory receptors and ligands beyond PD-1, PD-L1 and CTLA-4: breakthroughs or backups. Nat. Immunol. 20, 1425–1434. 10.1038/s41590-019-0512-0 31611702

[B2] Bae W. K. Lee B. C. Kim H. J. Lee J. J. Chung I. J. Cho S. B. (2022). A phase I study of locoregional high-dose autologous natural killer cell therapy with hepatic arterial infusion chemotherapy in patients with locally advanced hepatocellular carcinoma. Front. Immunol. 13, 879452. 10.3389/fimmu.2022.879452 35720374 PMC9202498

[B3] Bagchi S. Yuan R. Engleman E. G. (2021). Immune checkpoint inhibitors for the treatment of cancer: clinical impact and mechanisms of response and resistance. Annu. Rev. Pathol. 16, 223–249. 10.1146/annurev-pathol-042020-042741 33197221

[B4] Brahmer J. R. Abu-Sbeih H. Ascierto P. A. Brufsky J. Cappelli L. C. Cortazar F. B. (2021). Society for Immunotherapy of Cancer (SITC) clinical practice guideline on immune checkpoint inhibitor-related adverse events. J. Immunother. Cancer, 9. 10.1136/jitc-2021-002435 PMC8237720 34172516

[B5] Bray F. Laversanne M. Sung H. Ferlay J. Siegel R. L. Soerjomataram I. (2024). Global cancer statistics 2022: GLOBOCAN estimates of incidence and mortality worldwide for 36 cancers in 185 countries. CA Cancer J. Clin. 74, 229–263. 10.3322/caac.21834 38572751

[B6] Cao S. (2023). Clinical efficacy observation of Yangzheng Xiaoji Capsules Combined with camrelizumab and apatinib in the treatment if middle and advanced hepatocellular carcinoma. Master's thesis from Anhui University of Traditional Chinese Medicine.

[B7] Cao W. Chen H. D. Yu Y. W. Li N. Chen W. Q. (2021). Changing profiles of cancer burden worldwide and in China: a secondary analysis of the global cancer statistics 2020. Chin. Med. J. Engl. 134, 783–791. 10.1097/CM9.0000000000001474 33734139 PMC8104205

[B8] Cao C. Liu Z. Zhang X. Chen K. (2024). A clinical study on the effect of Wenyang Fuzheng Formula combined with PD-1 immunotherapy on the microenvironment of Yang deficiency type of liver cancer. Labeled Immunoassays and Clin Med 31, 1042–1048.

[B9] Chai H. (2023). Efficacy and safety of Yiqi Tongluo Jiedu decoction combined with chemotherapy and immunotherapy for advanced lung adenocarcinoma. Master's thesis Anhui Univ. Traditional Chin. Med.

[B10] Chen T. (2022). Clinical Study of astragalus polysaccharide injection combined with camrelizumab and apatinib in the treatment of Qi-deficiency lung cancer. Master's thesis from Tianjin University of Traditional Chinese Medicine.

[B11] Chen F. Kolben T. Meister S. Czogalla B. Kolben T. M. Hester A. (2022). The role of resveratrol, Sirtuin1 and RXRα as prognostic markers in ovarian cancer. Arch. Gynecol. Obstet. 305, 1559–1572. 10.1007/s00404-021-06262-w 34870752 PMC9166836

[B12] Chen G. Zhang H. Sun H. Ding X. Liu G. Yang F. (2023a). Bufalin targeting BFAR inhibits the occurrence and metastasis of gastric cancer through PI3K/AKT/mTOR signal pathway. Apoptosis 28, 1390–1405. 10.1007/s10495-023-01855-z 37253905

[B13] Chen L. Zheng X. Huang H. Feng C. Wu S. Chen R. (2023b). Cordycepin synergizes with CTLA-4 blockade to remodel the tumor microenvironment for enhanced cancer immunotherapy. Int. Immunopharmacol. 124, 110786. 10.1016/j.intimp.2023.110786 37611443

[B14] Cheng L. Liu W. Zhong C. Ni P. Ni S. Wang Q. (2021). Remodeling the homeostasis of pro- and anti-angiogenic factors by Shenmai injection to normalize tumor vasculature for enhanced cancer chemotherapy. J. Ethnopharmacol. 270, 113770. 10.1016/j.jep.2020.113770 33388426

[B15] Cunningham M. Gupta R. Butler M. (2024). Checkpoint inhibitor hepatotoxicity: pathogenesis and management. Hepatology 79, 198–212. 10.1097/HEP.0000000000000045 36633259

[B16] Ding H. Chen Z. Yu H. Feng Z. Shi L. (2024). Efficacy of Fried Licorice Decoction Plus-minus combined with PD-1 for advanced non-small cell lung cancer with Qi-Yin deficiency. Chin. J. Clin. Oncol. 51, 461–466.

[B17] Dou Q. (2022). “Clinical Study of Jianpi Huatan Quyu recipe combined with nab-paclitaxel,” in Platinum and camrelizumab in the treatment of advanced esophageal squamous cell carcinoma. Master's thesis from. Nanjing University of Chinese Medicine.

[B18] Du N. Liu Q. (2022). The efficacy and immune function of the combination of spleen tonifying and lung tonifying therapy and PD-1 monoclonal antibody in neoadjuvant chemotherapy for advanced non-small cell lung cancer. J. Clin. Res. 39, 294–297.

[B19] Egger M. Davey Smith G. Schneider M. Minder C. (1997). Bias in meta-analysis detected by a simple, graphical test. Bmj 315, 629–634. 10.1136/bmj.315.7109.629 9310563 PMC2127453

[B20] Fang H. (2023). From the perspective of immune regulation to explore the eight flavor soup effect on immunocombined with chemotherapy. Master's thesis from Anhui University of Traditional Chinese Medicine.

[B21] Gao W. Wang X. Zhou Y. Wang X. Yu Y. (2022). Autophagy, ferroptosis, pyroptosis, and necroptosis in tumor immunotherapy. Signal Transduct. Target Ther. 7, 196. 10.1038/s41392-022-01046-3 35725836 PMC9208265

[B22] Higgins J. P. Thompson S. G. Deeks J. J. Altman D. G. (2003). Measuring inconsistency in meta-analyses. Bmj 327, 557–560. 10.1136/bmj.327.7414.557 12958120 PMC192859

[B23] Higgins J. P. Altman D. G. Gøtzsche P. C. Jüni P. Moher D. Oxman A. D. (2011). The Cochrane Collaboration's tool for assessing risk of bias in randomised trials. Bmj 343, d5928. 10.1136/bmj.d5928 22008217 PMC3196245

[B24] Hu M. Yao W. Shen Q. (2022). Advances and challenges of immunocheckpoint inhibitors in the treatment of primary liver cancer. Front. Genet. 13, 1005658. 10.3389/fgene.2022.1005658 36246617 PMC9561712

[B25] Huang X. N. Zhu C. Li Y. Jin C. (2023). Weidiao-3 mixture improves the clinical efficacy of immunotherapy for advanced gastric cancer by regulating intestinal flora. Acta Acad. Med. Sin. 45, 581–590. 10.3881/j.issn.1000-503X.15496 37654138

[B26] Huang P. P. Shu Y. Sun H. Chen T. Jiang Y. Ma M. (2024). Clinical Study on the use of sini decoction in the treatment of 30 cases of advanced primary liver cancer with Yang deficiency. Jiangsu J. Traditional Chin. Med. 56, 39–42.

[B27] Jiang J. Fang Y. (2024). Application value of Buzhong Yiqi decoction in immunotherapy of NSCLC with negative driver gene. Int. Med. Health Guid. News 2, 305–309.

[B28] Jiang Z. B. Huang J. M. Xie Y. J. Zhang Y. Z. Hang Y. Z. Chang C. (2020). Evodiamine suppresses non-small cell lung cancer by elevating CD8(+) T cells and downregulating the MUC1-C/PD-L1 axis. J. Exp. Clin. Cancer Res. 39, 249. 10.1186/s13046-020-01741-5 33208183 PMC7677782

[B29] Li J. Li L. Liu R. Lin H. S. (2012). Establishing Chinese medicine characteristic tumor response evaluation system is the key to promote internationalization of Chinese medicine oncology. Chin. J. Integr. Med. 18, 730–736. 10.1007/s11655-012-1254-0 22965698

[B30] Lin X. T. (2023). Study of shenqi Fuzheng Injection combined camrelizumab with chemotherapy in the treatment of advanced non-small cell lung cancer. Master's thesis from Guangzhou University of Chinese Medicine.

[B31] Lin B. Q. Zeng L. Li S. (2022). Effect of Fuzheng Jiandu formula regulating Thl/Th2 immune balance on immunotherapy of advanced lung cancer and Study on its mechanism. Shanxi J TCM. 38, 18–20.

[B32] Liu T. Wang J. (2024). The effect of xiaoaiping combined with camrelizumab on Serum VEGF and tumor markers in advanced esophageal cancer. China Mod. Dr. 62, 71–73.

[B33] Liu W. Xia L. (2024). Efficacy observation of Fuzheng Sanjie Formula, camrelizumab and apatinib on advanced metastatic esophageal squamous carcinoma with syndrome of zheng-deficiency-phlegm-stasis. Clin. J. Traditional Chin. Med. 36, 939–944.

[B34] Liu F. L. Ma L. Song R. Huang Y. (2022). The effect of modified Xiaoyan decoction combined with PD-1 monoclonal antibody in neoadjuvant therapy for advanced NSCLC and its significance on immune function. Chin. J. Lung Dis. 15, 79–81.

[B35] Luo Y. (2023). Observation of the therapeutic effect of Jianpi Bufei formula combined with PD-1 monoclonal antibody and DP regimen in the treatment of non-small cell lung cancer. J. Sichuan Traditional Chin. Med. 41, 98–101.

[B36] Luo D. Wan X. Liu J. Tong T. (2018). Optimally estimating the sample mean from the sample size, median, mid-range, and/or mid-quartile range. Stat. Methods Med. Res. 27, 1785–1805. 10.1177/0962280216669183 27683581

[B37] Marei H. E. Hasan A. Pozzoli G. Cenciarelli C. (2023). Cancer immunotherapy with immune checkpoint inhibitors (ICIs): potential, mechanisms of resistance, and strategies for reinvigorating T cell responsiveness when resistance is acquired. Cancer Cell Int. 23, 64. 10.1186/s12935-023-02902-0 37038154 PMC10088229

[B38] Moher D. Liberati A. Tetzlaff J. Altman D. G. (2009). Preferred reporting items for systematic reviews and meta-analyses: the PRISMA statement. PLoS Med. 6, e1000097. 10.1371/journal.pmed.1000097 19621072 PMC2707599

[B39] Qu X. P. (2023). Observation on the clinical efficacy of Bufei decoction combined with chemotherapy and immunotherapy for advanced non-small cell lung cancer with lung Qi deficiency syndrome. Master's thesis Hubei Univ. Chin. Med.

[B40] Ramos-Casals M. Brahmer J. R. Callahan M. K. Flores-Chávez A. Keegan N. Khamashta M. A. (2022). Immune-related adverse events of checkpoint inhibitors. Nat. Rev. Dis. Prim. 6, 38. 10.1038/s41572-020-0160-6 32382051 PMC9728094

[B41] Ren W. Liang P. Ma Y. Sun Q. Pu Q. Dong L. (2021). Research progress of traditional Chinese medicine against COVID-19. Biomed. Pharmacother. 137, 111310. 10.1016/j.biopha.2021.111310 33761591 PMC7857050

[B42] Rui R. Zhou L. He S. (2023). Cancer immunotherapies: advances and bottlenecks. Front. Immunol. 14, 1212476. 10.3389/fimmu.2023.1212476 37691932 PMC10484345

[B43] Schmidt F. L. Oh I. S. Hayes T. L. (2009). Fixed-*versus* random-effects models in meta-analysis: model properties and an empirical comparison of differences in results. Br. J. Math. Stat. Psychol. 62, 97–128. 10.1348/000711007X255327 18001516

[B44] Seto T. Sam D. Pan M. (2019). Mechanisms of primary and secondary resistance to immune checkpoint inhibitors in cancer. Med. Sci. (Basel). 7. 30678257 10.3390/medsci7020014 PMC6410194

[B45] Shi J. Luo D. Wan X. Liu Y. Liu J. Bian Z. (2023). Detecting the skewness of data from the five-number summary and its application in meta-analysis. Stat. Methods Med. Res. 32, 1338–1360. 10.1177/09622802231172043 37161735

[B46] Upadhyay R. Elguindy A. N. M. Salts L. Donovan K. Sengupta S. Wang K. (2025). Boswellia Serrata for cerebral radiation necrosis after radiosurgery for brain metastases. Int. J. Radiat. Oncol. Biol. Phys. 122, 1282–1291. 10.1016/j.ijrobp.2025.02.016 39993542

[B47] Vafaei S. Zekiy A. O. Khanamir R. A. Zaman B. A. Ghayourvahdat A. Azimizonuzi H. (2022). Combination therapy with immune checkpoint inhibitors (ICIs); a new frontier. Cancer Cell Int. 22, 2. 10.1186/s12935-021-02407-8 34980128 PMC8725311

[B48] Wan X. Wang W. Liu J. Tong T. (2014). Estimating the sample mean and standard deviation from the sample size, median, range and/or interquartile range. BMC Med. Res. Methodol. 14, 135. 10.1186/1471-2288-14-135 25524443 PMC4383202

[B49] Wang X. Y. (2021). Clinical observation of shenqi Yifei decoction in treating advanced non-small cell lung cancer with deficiency of Qi and yin. Master's thesis from Shandong University of Traditional Chinese Medicine.

[B50] Wang Q. (2023a). Clinical observation of modified Liujunzi decoction combined with tirelizumab an chemotherapy in the first-line treatment of advanced lung squamous cell carcinoma. Master's thesis Anhui Univ. Traditional Chin. Med.

[B51] Wang X. H. (2023b). Clinical and mechanism Study of dushen decoction combined with chemotherapy and immunotherapy for advanced lung squamous cell carcinoma. Master's thesis Shandong Univ. Traditional Chin. Med.

[B52] Wang X. F. (2024). Clinical observation of the combination of Jianpi Huatan prescription with PD-1 inhibitor and the SOX regimen in the treatment of advanced gastric cancer with spleen deficiency combined with phlegm and stasis syndrome. Master's thesis from Nanjing University of Chinese Medicine.

[B53] Wang K. Chen Q. Shao Y. Yin S. Liu C. Liu Y. (2021a). Anticancer activities of TCM and their active components against tumor metastasis. Biomed. Pharmacother. 133, 111044. 10.1016/j.biopha.2020.111044 33378952

[B54] Wang S. Fu J. L. Hao H. F. Jiao Y. N. Li P. P. Han S. Y. (2021b). Metabolic reprogramming by traditional Chinese medicine and its role in effective cancer therapy. Pharmacol. Res. 170, 105728. 10.1016/j.phrs.2021.105728 34119622

[B55] Wang Z. Wang Y. Gao P. Ding J. (2023a). Immune checkpoint inhibitor resistance in hepatocellular carcinoma. Cancer Lett. 555, 216038. 10.1016/j.canlet.2022.216038 36529238

[B56] Wang L. Wang M. Kan C. (2023b). Effects of Jianpi Huatan xiaoying decoction on autoimmunity, swallowing function and Serum MIP-1, sIL-2R and S-TKl in patients with thyroid dysfunction induced by immunotherapy of lung cancer. Eval. analysis drug-use Hosp. China 23, 935–939.

[B57] Wang X. Chen H. Hong W. Yang Y. Wang S. Wang C. (2024). Qingfei tiaoqi decoction can reduce the immune checkpoint inhibitor-related adverse events in lung cancer patients by regulating the intestinal flora structure. Mod. Oncol. 32, 890–895.

[B58] Xiang Y. Guo Z. Zhu P. Chen J. Huang Y. (2019). Traditional Chinese medicine as a cancer treatment: modern perspectives of ancient but advanced science. Cancer Med. 8, 1958–1975. 10.1002/cam4.2108 30945475 PMC6536969

[B59] Xiao H. Q. Li X. Xie J. Xie S. (2022). Combination of traditional Chinese medicine decoction and immunotherapy for advanced lung cancer in elderly patients research on the application effect in patients. Electron. J. Clin. Med. Literature 48, 52–54.

[B60] Xu W. W. (2023). Clinical observation on the third-line treatment of advanced gastric cancer with Wenyang Tongluo Formula combined with PD-1 inhibitor, apatinib and tegafur. Master's thesis from Anhui University of Traditional Chinese Medicine.

[B61] Yang H. (2023). The clinical research of the treatment on NSCLC patients with Yanghe decoction combined with immune checkpoint inhibitors. Master's thesis from Jiangxi University of Chinese Medicine.

[B62] Yao S. W. (2023). Clinical observation of qigui Buxue syrup on cancer-related fatigue after immunotherapy for non-small cell lung cancer of lung-spleen deficiency type. Master's thesis from Chengde Medical University.

[B63] Ye Z. (2022). Clinical observation of modified Wumei decoction combined with chemotherapy and immunotherapy for mixed cold and heat esophageal cancer. Master's thesis from Henan University of Chinese Medicine.

[B64] Ye J. H. Fang Z. (2022). Efficacy observation of Jianpi Huoxue formula and camrelizumab and Lenvatinib on liver cancer. Shanxi J TCM. 38, 35–37.

[B65] Yu Y. X. Wang S. Liu Z. N. Zhang X. Hu Z. X. Dong H. J. (2023a). Traditional Chinese medicine in the era of immune checkpoint inhibitor: theory, development, and future directions. Chin. Med. 18, 59. 10.1186/s13020-023-00751-7 37210537 PMC10199665

[B66] Yu D. He S. Shen J. Hu N. Cai Y. Cao T. (2023b). Clinical observation of ruyi Jinhuang Powder combined with ICIs in the treatment of advanced liver cancer complicated with dampness and heat syndrome of liver and gallbladder. China Pharm. 34, 1488–1492.

[B67] Zeng Y. (2018). Advances in mechanism and treatment strategy of cancer. Cell Mol. Biol. (Noisy-le-grand) 64, 1–3. 29808792

[B68] Zhang Z. (2022). Clinical observation of jianpi chuji formula combined with immune-checkpoint inhibitors in the treatment of advanced non-small cell lung cancer. Master's thesis from Chengde Medical University.

[B69] Zhang J. (2023a). Clinical Study of shenqi Fuzheng injection combined with endostar and PD-1 inhibitor in the treatment of driver gene negative NSCLC. Master's thesis from Tianjin University of traditional Chinese medicine.

[B70] Zhang L. L. (2023b). Efficacy and safety of hepatoma I formula plus minus plus and minus plus PD-1 inhibitor in the treatment of advanced primary hepatoma. Master's thesis from Gansu University of Chinese Medicine.

[B71] Zhang Y. Lou Y. Wang J. Yu C. Shen W. (2020). Research status and molecular mechanism of the traditional Chinese medicine and antitumor therapy combined strategy based on tumor microenvironment. Front. Immunol. 11, 609705. 10.3389/fimmu.2020.609705 33552068 PMC7859437

[B72] Zhang J. Huang D. Saw P. E. Song E. (2022). Turning cold tumors hot: from molecular mechanisms to clinical applications. Trends Immunol. 43, 523–545. 10.1016/j.it.2022.04.010 35624021

[B73] Zhang Y. Y. Han F. Cao Y. Zhang Y. Zhang X. Hu J. (2023). Effect of Bushen Jiedu Sanjie recipe on Immune Micro-environment of patients with advanced colorectal cancer based on PD-1/PD-L1 pathway. Pract. Pharm. And Clin. Remedies 26, 306–310.

[B74] Zhao Z. D. (2024). Clinical observation on the therapeutic effect of Yiqi shengmai formula combined with herbal moxibustion and immunotherapy on advanced non-small cell lung cancer with Qi and yin deficiency type. Master's thesis from Chengde Medical University.

[B75] Zhao J. L. Xu H. Yu X. Lu H. Wang X. Chen L. (2023). Clinical effect of peitu zishen decoction combined with immunotherapy in the treatment of advanced non-small. Cell Lung Cancer 61, 80–83.

[B76] Zhong X. T. (2023). Clinical observation on the therapeutic effect of Fuhe Beihua Formula combined with Karelizumab and apatinib in the treatment of advanced liver cancer with liver depression and spleen deficiency syndrome after TACE surgery. Master's thesis from Guangxi University of Chinese Medicine.

[B77] Zhou B. (2023). Clinical effect of shenqi busui decoction in treatment of Qi-blood deficiency-type advanced non-small cell lung cancer without driver mutations:an analysis of 45 cases. Master's thesis Hunan J. Traditional Chin. Med.

[B78] Zhou D. Xie L. (2024). Clinical Study of guizhifuling pill combined with PD-1 in the treatment of advanced ovarian cancer. Electron. JournaI Pract. GynecoIogical EndocrinoIogy 11, 68–70.

[B79] Zhou P. Gao Y. Kong Z. Wang J. Si S. Han W. (2024). Immune checkpoint inhibitors and acute kidney injury. Front. Immunol. 15, 1353339. 10.3389/fimmu.2024.1353339 38464524 PMC10920224

[B80] Zhu S. Y. (2024). Study on the clinical and experimental of Fuzheng Guben Decoction on immune maintenance in advanced lung cancer. Doctor’s thesis from Changchun University of Chinese Medicine.

[B81] Zhu C. Li K. Peng X. X. Yao T. J. Wang Z. Y. Hu P. (2022). Berberine a traditional Chinese drug repurposing: its actions in inflammation-associated ulcerative colitis and cancer therapy. Front. Immunol. 13, 1083788. 10.3389/fimmu.2022.1083788 36561763 PMC9763584

