# Supplementary figures and images for: Efficacy and safety of traditional Chinese medicine (TCM) combined with immune checkpoint inhibitors (ICIs) for the treatment of cancer: a systematic review and meta-analysis
Source: Front Pharmacol. 2025 Oct 31;16:1661503. doi: 10.3389/fphar.2025.1661503 (PMC12615493; doi:10.3389/fphar.2025.1661503)

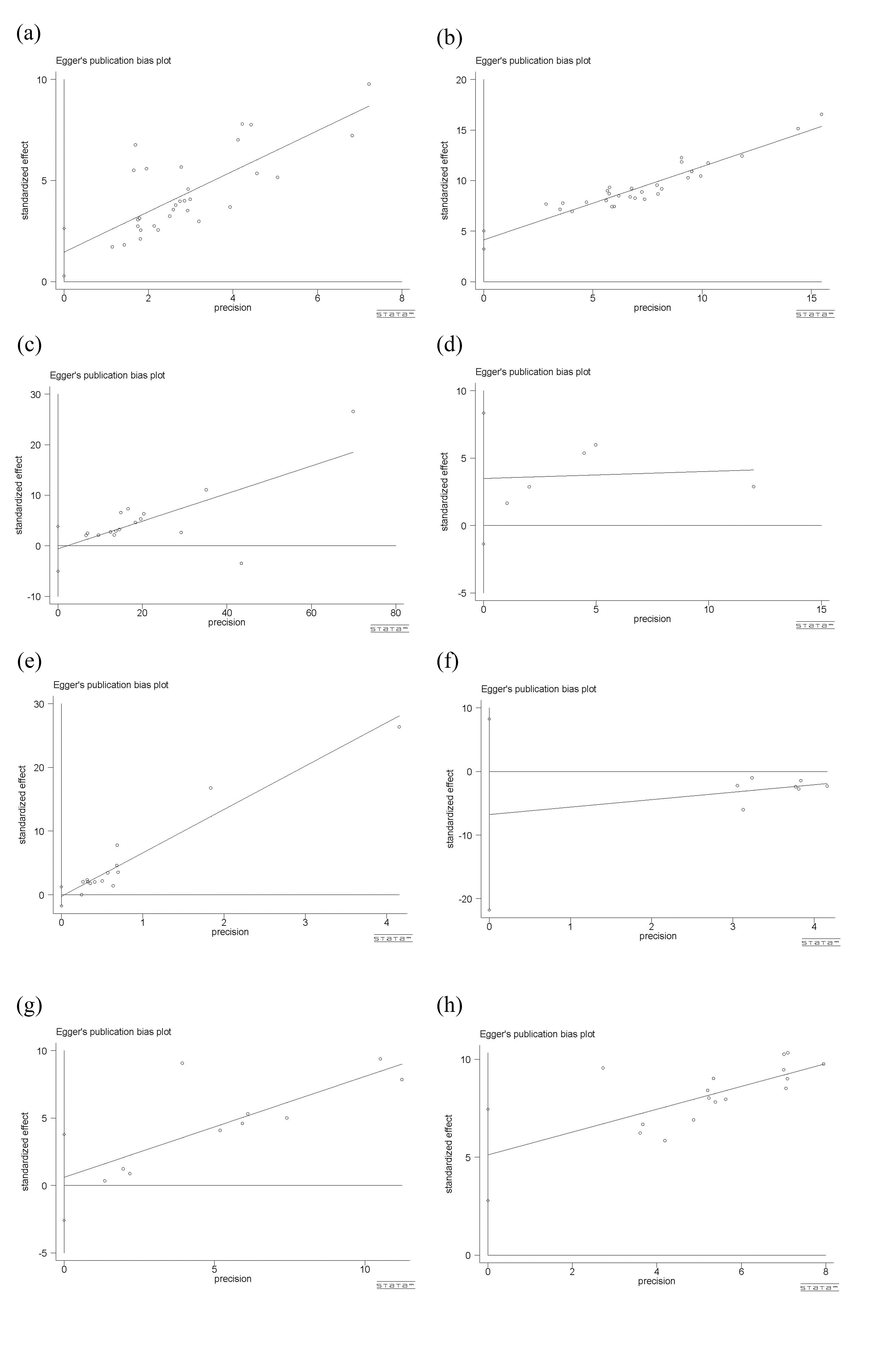

Supplement: Supplementary file 1 [file Supplementaryfile8.jpeg]

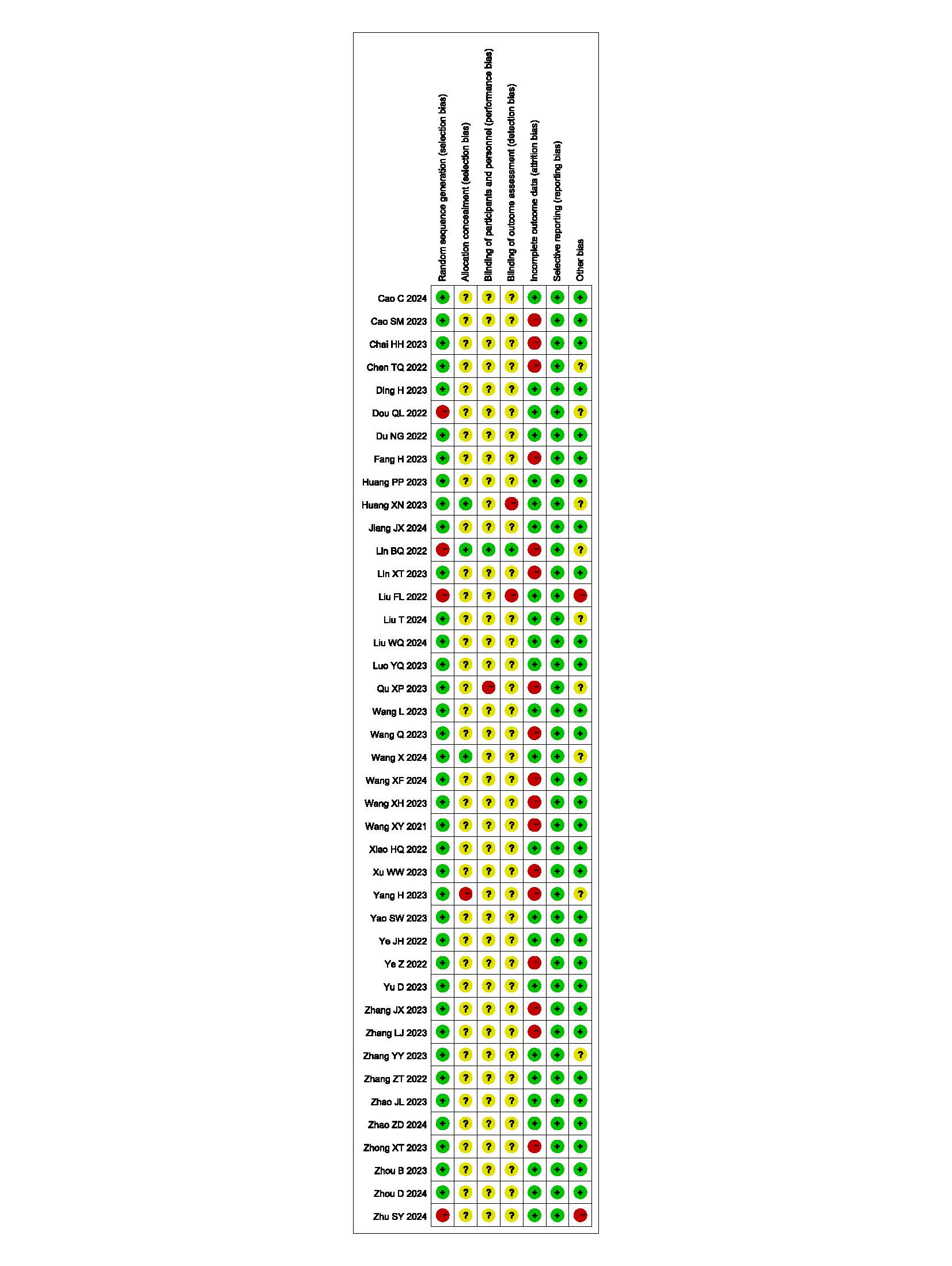

Supplement: Supplementary file 5 [file Supplementaryfile5.jpeg]

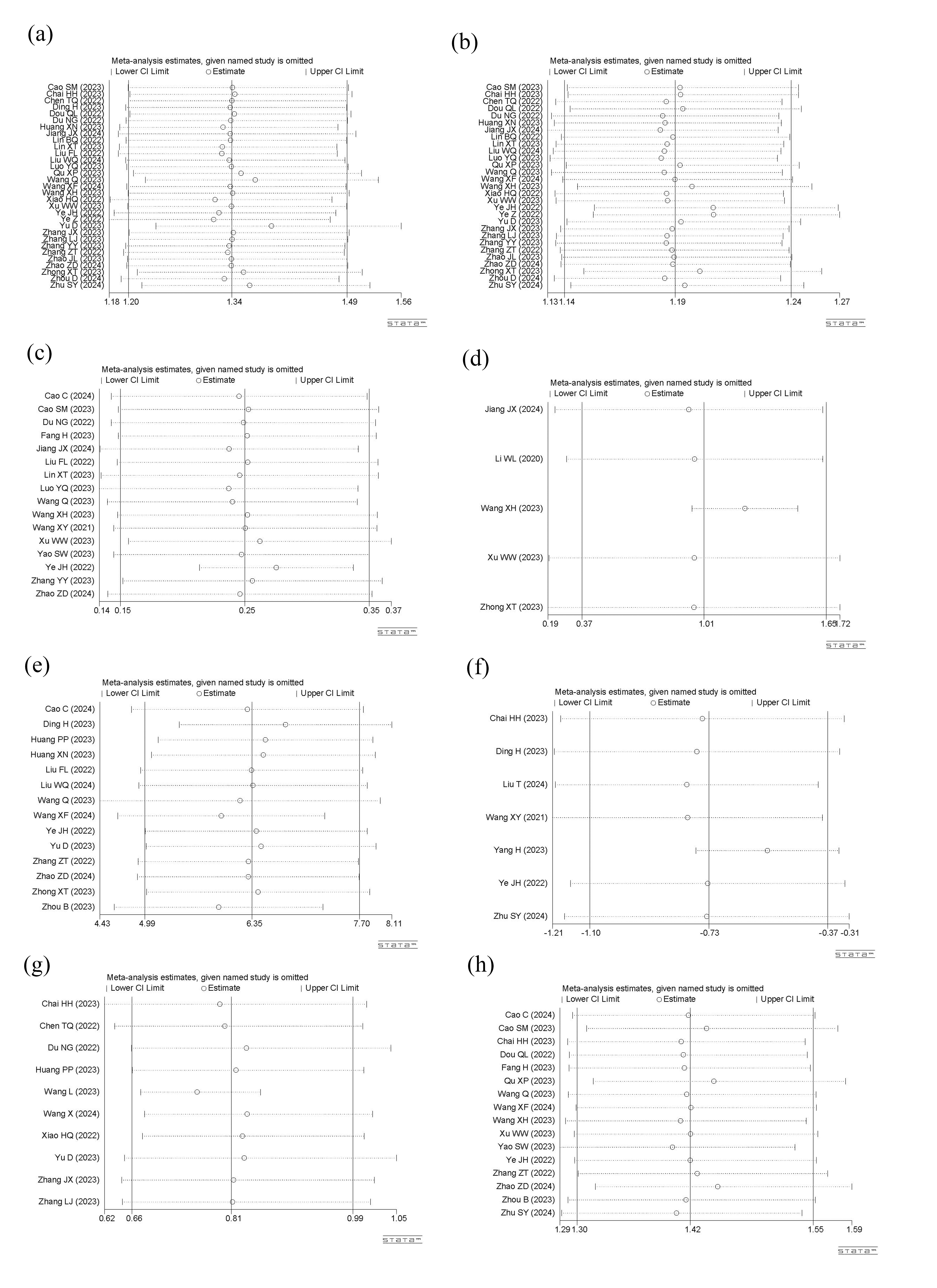

Supplement: Supplementary file 6 [file Supplementaryfile9.jpeg]
