# Supplementary material for: Efficacy and safety of traditional Chinese medicine (TCM) combined with immune checkpoint inhibitors (ICIs) for the treatment of cancer: a systematic review and meta-analysis
Source: Front Pharmacol. 2025 Oct 31;16:1661503. doi: 10.3389/fphar.2025.1661503 (PMC12615493; doi:10.3389/fphar.2025.1661503)
Supplement: Supplementary file 2 [file Supplementaryfile1.pdf]

NIHR | National Institute for Health and Care Research

PROSPERO
International prospective register of systematic reviews

Efficacy and safety of traditional Chinese medicine combined with immunotherapy for the treatment of cancer: A Systematic Review and Meta-Analysis

KE YANI, Yuyan Pan

To enable PROSPERO to focus on COVID-19 submissions, this registration record has undergone basic automated checks for eligibility and is published exactly as submitted. PROSPERO has never provided peer review, and usual checking by the PROSPERO team does not endorse content. Therefore, automatically published records should be treated as any other PROSPERO registration. Further detail is provided here.

Citation
KE YANI, Yuyan Pan. Efficacy and safety of traditional Chinese medicine combined with immunotherapy for the treatment of cancer: A Systematic Review and Meta-Analysis. PROSPERO 2024 Available from https://www.crd.york.ac.uk/PROSPERO/view/CRD42024582055

REVIEW TITLE AND BASIC DETAILS

Review title
Efficacy and safety of traditional Chinese medicine combined with immunotherapy for the treatment of cancer: A Systematic Review and Meta-Analysis

Review objectives
Can traditional Chinese medicine enhance the anti-tumor effect of immunotherapy?

SEARCHING AND SCREENING

Searches
Database: PubMed, EMBASE, Cochrane Library, ClinicalTrials.gov, CNKI, WANFANG, CBM and grey literature
Publication period: up to August, 2024
Language: English and Chinese
Search strategy: (“Traditional Chinese Medicine” OR “herbal medicine” OR “TCM” OR “herbs”) AND (“immunotherapy” OR “immune checkpoint inhibitors” OR

“antitumor immunity” OR “PD-1” OR “PD-L1” OR “programmed cell death protein 1” OR “programmed death ligand 1”)

Study design

Randomized Controlled Trial

ELIGIBILITY CRITERIA

Condition or domain being studied

According to statistics, nearly one sixth of deaths every year are caused by cancer, which seriously threatens human health. The global burden of cancer incidence rate and mortality is rapidly increasing. The anti-cancer efficacy of traditional treatment methods such as surgical resection, chemotherapy, and radiotherapy, which are commonly used in clinical practice, is not ideal. Immunotherapy has become a new type of anti-tumor method and hotspot after traditional treatment methods. The mechanism is mainly guided by immunological theory, using methods such as redirection and gene programming to activate the human immune system, target tumor cells, and treat tumors.

In recent years, traditional Chinese medicine (TCM) plays a significant role in improving tumor symptoms, alleviating discomfort after radiotherapy and chemotherapy, improving patients' quality of life, and prolonging survival time. TCM has the characteristic of "supporting the body and dispelling evil", which has demonstrated advantages in reversing drug resistance, regulating the tumor microenvironment, reducing toxic side effects, and improving tumor control and remission rates. The use of TCM to treat tumors in clinical practice has also become a preferred choice for the public. Therefore, exploring the efficacy and safety of TCM combined with immunotherapy in combating tumors is a direction for future research.

Population

Inclusion criteria: adults (aged 18 years or older); patients must be diagnosed with cancer; One group received traditional Chinese medicine and immunotherapy, while the other group received immunotherapy; Randomized Controlled Trial.

Intervention(s) or exposure(s)

Individuals with cancer, different kinds of cancer, different kinds of immunotherapy/TCM, different outcome measures

Comparator(s) or control(s)

Cancer patients receiving immunotherapy
Healthy people without cancer

OUTCOMES TO BE ANALYSED

Main outcomes

Cr, Pr, SD, PD, KPS, adverse effects, etc.

Additional outcomes

PFS, TTP, etc.

DATA COLLECTION PROCESS

Data extraction (selection and coding)

Two members are responsible for extracting data, including the first author’ s name, publication time, country of origin, quality score, number of patients in each group, basic information of each group (age, gender, etc.), diagnostic methods, treatment methods, efficacy evaluation, safety evaluation, etc. If there are any issues, it will be decided by the third member. When the information is incomplete, we will contact the author team of the article to obtain relevant information.

Risk of bias (quality) assessment

All quality assessments are conducted independently by two members. The disputed part is left to the third member to decide. The assessment of publication bias is based on Eggar's Test and funnel plot. Sensitivity analysis can evaluate the stability of the results.

PLANNED DATA SYNTHESIS

Strategy for data synthesis

We will use Review Manager 5.3 and Stata 12 software to analyze all the data. The heterogeneity among studies is determined by p-values and I^2. When the heterogeneity is relatively low, a fixed effects models is more suitable. If the heterogeneity is high, a random effects model will be chosen. In addition, subgroup analysis and meta-regression would be performed to further explore possible sources of heterogeneity.

Analysis of subgroups or subsets

Subgroups classified by different kind of cancer or by different ethnicities or by different treatment.

REVIEW AFFILIATION, FUNDING AND PEER REVIEW

Review team members

- Miss KE YANI, Zhejiang Chinese Medical University
- Dr Yuyan Pan,

Review affiliation

Zhejiang Chinese Medical University

Funding source

None

TIMELINE OF THE REVIEW

Review timeline

Start date: 15 September 2024. End date: 15 March 2025

Date of first submission to PROSPERO

21 August 2024

Date of registration in PROSPERO
01 September 2024

CURRENT REVIEW STAGE

Publication of review results
The intention is not to publish the review once completed.

Stage of the review at this submission

Review stage	Started	Completed
Pilot work		
Formal searching/study identification		
Screening search results against inclusion criteria		
Data extraction or receipt of IP		
Risk of bias/quality assessment		
Data synthesis		

Review status
The review is currently planned or ongoing.

ADDITIONAL INFORMATION

PROSPERO version history

- Version 1.1 published on 01 Sep 2024
- Version 1.0 published on 01 Sep 2024

Review conflict of interest
None known

Country
China

Medical Subject Headings
Humans; Immunotherapy; Medicine, Chinese Traditional; Neoplasms

Disclaimer
The content of this record displays the information provided by the review team. PROSPERO does not peer review registration records or endorse their content.

PROSPERO accepts and posts the information provided in good faith; responsibility for record content rests with the review team. The owner of this record has affirmed that the information provided is truthful and that they understand that deliberate provision of inaccurate information may be construed as scientific misconduct.

PROSPERO does not accept any liability for the content provided in this record or for its use. Readers use the information provided in this record at their own risk.

Any enquiries about the record should be referred to the named review contact
