# Supplementary material for: Efficacy and safety of traditional Chinese medicine (TCM) combined with immune checkpoint inhibitors (ICIs) for the treatment of cancer: a systematic review and meta-analysis
Source: Front Pharmacol. 2025 Oct 31;16:1661503. doi: 10.3389/fphar.2025.1661503 (PMC12615493; doi:10.3389/fphar.2025.1661503)
Supplement: Supplementary file 7 [file Supplementaryfile2.docx]

| Databases | Search terms | Number of records |
| --- | --- | --- |
| Pubmed | Search：(('Traditional Chinese Medicine')) OR ('herbal medicine') OR ('TCM') OR ('herbs') OR ('TCM'[MeSH Terms])) AND (('immunotherapy') OR ('immune checkpoint inhibitors') OR ('antitumor immunity') OR ('PD-1') OR ('PD-L1') OR ('programmed cell death protein 1') OR ('programmed death ligand 1') OR ('ICIs') OR ('ICIs'[MeSH Terms]) OR ('immunotherapy'[MeSH Terms]) OR ('PD-1'[MeSH Terms])) | 780 |
| Cochrane Library | #1MeSH descriptor:[ICIs] explode all trees  #2'ICIs'  #3'immunotherapy'  #4'immune checkpoint inhibitors'  #5'PD-1'  #6MeSH descriptor:[immunotherapy] explode all trees  #7'PD-L1'  #8'programmed cell death protein 1'  #9MeSH descriptor:[PD-1] explode all trees  #10'programmed death ligand 1'  #11'antitumor immunity'  #12 #1 or #2 or #3 or #4 or #5 or #6 or #7 or #8 or #9 or #10 or #11  #13MeSH descriptor:[TCM] explode all trees  #14'TCM'  #15'Traditional Chinese Medicine'  #16'herbal medicine'  #17'herbs'  #18 #13 or #14 or #15 or #16 or #17  #19 #12 and #18 | 118 |
| EMBASE | #1'ICIs'/exp  #2'ICIs'  #3'immunotherapy'  #4'immune checkpoint inhibitors'  #5'PD-1'  #6'PD-L1'  #7'programmed cell death protein 1'  #8'immunotherapy'/exp  #9'programmed death ligand 1'  #10'antitumor immunity'  #11'PD-1'/exp  #12 #1 or #2 or #3 or #4 or #5 or #6 or #7 or #8 or #9 or #10 or #11  #13'TCM'/exp  #14'TCM'  #15'Traditional Chinese Medicine'  #16'herbal medicine'  #17'herbs'  #18 #13 or # 14 or #15 or #16 or #17  #19 #12 and #18 | 1941 |
| CNKI | (SU% ='中药' OR SU% ='中草药' OR SU% ='草药' OR SU% ='中医' OR SU% ='中医药' OR SU% ='TCM') AND (SU% ='PD-1' OR SU% ='PD-L1' OR SU% ='ICIs' OR SU% ='免疫治疗' OR SU% ='免疫检查点抑制治疗' OR SU% ='免疫疗法' OR SU% ='免疫药物') | 1595 |
| Wanfang | 题名或关键词：((中药 or 中草药 or 草药 or 中医 or 中医药 or TCM) and (PD-1 or PD-L1 or ICIs or 免疫治疗 or 免疫检查点抑制治疗 or 免疫疗法 or 免疫药物)) | 1232 |
| CBM | ("中药"[摘要] OR "中草药"[摘要] OR "草药"[摘要] OR "中医"[摘要] OR "中医药"[摘要] OR "草药"[摘要] OR "TCM"[摘要]) AND ("PD-1"[摘要] OR "PD-L1"[摘要] OR "ICIs"[摘要] OR "免疫治疗"[摘要] OR "免疫检查点抑制治疗"[摘要] OR "免疫疗法"[摘要] OR "免疫药物"[摘要]) | 1160 |
